# Differentiating phosphate-dependent and phosphate-independent systemic phosphate-starvation response networks in *Arabidopsis thaliana* through the application of phosphite

**DOI:** 10.1093/jxb/erv025

**Published:** 2015-02-19

**Authors:** Ricarda Jost, Made Pharmawati, Hazel R. Lapis-Gaza, Claudia Rossig, Oliver Berkowitz, Hans Lambers, Patrick M. Finnegan

**Affiliations:** ^1^School of Plant Biology, The University of Western Australia, Crawley (Perth), Western Australia, Australia; ^2^Biology Department, Faculty of Mathematics and Natural Sciences, Bukit Jimbaran Campus, Udayana University, Bali, Indonesia; ^3^School of Veterinary and Life Sciences, Murdoch University, Murdoch, Western Australia, Australia; ^4^Institute of Agriculture, The University of Western Australia, Crawley (Perth), Western Australia, Australia

**Keywords:** *Arabidopsis thaliana*, phosphate-starvation response, phosphate transport, phosphite, phosphonate, phosphorous acid, phosphorus signalling networks, PSR genes, transcriptional regulation.

## Abstract

Phosphite is a less oxidized form of phosphorus than phosphate. Phosphite is considered to be taken up by the plant through phosphate transporters. It can mimic phosphate to some extent, but it is not metabolized into organophosphates. Phosphite could therefore interfere with phosphorus signalling networks. Typical physiological and transcriptional responses to low phosphate availability were investigated and the short-term kinetics of their reversion by phosphite, compared with phosphate, were determined in both roots and shoots of *Arabidopsis thaliana*. Phosphite treatment resulted in a strong growth arrest. It mimicked phosphate in causing a reduction in leaf anthocyanins and in the expression of a subset of the phosphate-starvation-responsive genes. However, the kinetics of the response were slower than for phosphate, which may be due to discrimination against phosphite by phosphate transporters PHT1;8 and PHT1;9 causing delayed shoot accumulation of phosphite. Transcripts encoding PHT1;7, lipid-remodelling enzymes such as SQD2, and phosphocholine-producing NMT3 were highly responsive to phosphite, suggesting their regulation by a direct phosphate-sensing network. Genes encoding components associated with the ‘PHO regulon’ in plants, such as *At4*, *IPS1*, and *PHO1;H1*, generally responded more slowly to phosphite than to phosphate, except for *SPX1* in roots and *MIR399d* in shoots. Two uncharacterized phosphate-responsive E3 ligase genes, *PUB35* and *C3HC4*, were also highly phosphite responsive. These results show that phosphite is a valuable tool to identify network components directly responsive to phosphate.

## Highlight

Phosphate transporters AtPHT1;8 and AtPHT1;9, but not AtPHT1;1, discriminate between phosphite and phosphate. Phosphate-starvation-responsive transcript profiles show altered kinetics with phosphite, hence allowing further dissection of phosphorus signalling networks.

## Introduction

Phosphite (H_2_PO_3_^–^, Phi) is a less oxidized form of phosphorus (P) than phosphate (H_2_PO_4_^–^, P_i_). Phi is highly water soluble and less prone than P_i_ to adsorb to soil particles, which makes it more accessible to plants (Ruthbaum and Baille, 1964). Phi competes with the essential macronutrient P_i_ for uptake by plants, most probably through both high- and low-affinity transport systems (d’arcy-Lameta and Bompeix, 1991; Danova-Alt *et al.*, 2008). Phi uptake is strongly and competitively inhibited in the presence of P_i_ (Pratt *et al.*, 2009). Within the plant, Phi can be translocated, and it preferentially accumulates in sink tissues (Nartvaranant *et al.*, 2004).

Phosphite was once abundant in the oceans, but it has been oxidized over time (Pasek *et al.*, 2013). Many microbes have retained the ability to oxidize Phi to P_i_, and even use it as a reducing agent, namely for sulphate reduction (Poehlein *et al.*, 2013). Plants, however, are not able to metabolize Phi (McDonald *et al.*, 2001). Instead, P-limited plants are highly sensitive to Phi and display toxicity symptoms such as leaf chlorosis and stunted growth (McDonald *et al.*, 2001; Ratjen and Gerendas, 2009; Thao and Yamakawa, 2009). Other detrimental effects caused by Phi are the arrest of primary root growth, yellowing of the leaf lamina of young leaves, and a patchy accumulation of anthocyanins in older leaves (Varadarajan *et al.*, 2002). Pratt *et al.* (2009) also showed that respiration rates declined upon Phi treatment of P-limited sycamore cells. It was recently found that the accumulation of Phi impacts on metabolism in *Arabidopsis thaliana*, leading to changes in the levels of several central metabolites (Berkowitz *et al.*, 2013).

Phi also triggers broad-spectrum resistance against pathogens with a (hemi)biotrophic lifestyle, such as oomycetes, fungi, and nematodes (Smillie *et al.*, 1989; Hofgaard *et al.*, 2010; Dias-Arieira *et al.*, 2013; Percival and Banks, 2014). Phi has been suggested to act as a priming agent of plant defence responses in a number of plant–pathogen interactions (Machinandiarena *et al.*, 2012; Massoud *et al.*, 2012; Dalio *et al.*, 2014). However, it is unclear how the primary recognition of Phi takes place, and which molecular pathways are altered within the plant subsequently to induce this primed state of heightened defence. Given that Phi is transported by P_i_ transporters, these primary molecular interactions could trigger changes in signal perception (Schothorst *et al.*, 2013).

Phi accumulates in both the cytosol and organelles, while the presence of P_i_ enhances Phi sequestration in the vacuole (Danova-Alt *et al.*, 2008). This is probably why plants with an adequate P status can tolerate moderate Phi exposure without visible toxicity symptoms (Thao and Yamakawa, 2009). Conversely, Phi inhibits the efflux of P_i_ from the vacuole, which could exacerbate P_i_-starvation symptoms (Pratt *et al.*, 2009) and lead to accelerated plant death (Singh *et al.*, 2003). Interestingly, the combined concentrations of Phi plus P_i_ within roots and shoots of *A. thaliana* were remarkably constant, regardless of their ratio in the growth medium, demonstrating that plants sense both P_i_ and Phi and adjust their uptake and allocation accordingly (Berkowitz *et al.*, 2013).

Due to its physical similarity to P_i_ and non-metabolizable nature, Phi has been used as a tool to understand P_i_-dependent signalling networks in plants. In several studies, Phi in fact seemed to mimic P_i_ effectively. *Brassica nigra* seedlings germinated on low-P_i_ media in the presence of high (1–10mM) Phi concentrations had reduced activation of P_i_-starvation-induced phospho*enol*pyruvate phosphatase and pyrophosphate-dependent phosphofructokinase compared with P-limited control plants (Carswell *et al.*, 1996). While Phi did not affect the total adenylate pool in P-limited *Brassica napus* suspension cells in the same way as P_i_, it did cause changes in the *in vivo* phosphorylation status of a number of proteins (Carswell *et al.*, 1997). In *A. thaliana*, Ticconi *et al.* (2001) observed that Phi prevented the induction of transcripts from the P_i_-starvation-responsive (PSR) genes *ACP5*, *At4*, and *PT2* upon 14 d exposure of P-sufficient seedlings to a medium lacking P_i_, but containing high concentrations of Phi. The same plants showed reduced *in vitro* activities of PSR ribonucleases RNS1 and RNS2 and of an acid phosphatase. Within 1 d of transfer of P-sufficient *A. thaliana* seedlings to a medium lacking P_i_, Phi suppressed the typical root hair formation and transcript accumulation of purple acid phosphatase *PAP1* and P_i_ transporters *PT1* and *PT2* that occur upon P_i_ withdrawal (Varadarajan *et al.*, 2002). Exposure of *A. thaliana* to Phi prevented not only PSR *MGD2* and *MGD3* expression, but also changes in glycerolipid profiles that accompany P-limited growth (Kobayashi *et al.*, 2006). In P-limited tomato seedlings, Phi mimicked P_i_ in promoting proteolytic turnover of purple acid phosphatases (Bozzo *et al.*, 2004). In rice, long-term exposure (5–7 d) to Phi suppressed the P_i_-starvation-induced expression of *OsIPS1* and *OsIPS2* (Hou *et al.*, 2005). In tobacco BY-2 cells, Phi caused the reversion of autophagic protein turnover triggered by P_i_ deprivation (Tasaki *et al.*, 2014).

The first evidence suggesting that Phi and P_i_ have discrete effects on P signalling networks came from work by Stefanovic *et al.* (2007), who showed that transcripts of *PHO1* and its close paralogue *PHO1;H1* differentially accumulated in plants treated with P_i_ or Phi. The PHR1-dependent induction of *PHO1;H1* under P-limiting conditions was attenuated by Phi, while the PHR1-independent induction of *PHO1* was not. This effect does not directly depend on the MYB transcription factor PHR1, because, unlike for *PHO1;H1*, the induction of another PHR1-regulated paralogue, *PHO1;H10*, was not affected by Phi (Ribot *et al.*, 2008). Interestingly, both *PHO1* and *PHO1;H1* transcripts were less abundant in the P-limited *pho2* mutant and more strongly induced in the P-limited *pdr2* mutant compared with those in the wild type (Stefanovic *et al.*, 2007). Disruption of the gene encoding endoplasmic reticulum (ER)-resident P_5_-type ATPase PDR2 affected local P_i_-sensing networks and heightened the sensitivity and amplitude of metabolic responses to P limitation (Ticconi *et al.*, 2004). The conditional *pdr2* short-root phenotype was reversible by Phi. These observations strongly suggest that Phi mimics P_i_ in local signalling networks, irrespective of the plant’s P status.

Studies have so far addressed the question of whether Phi can prevent the long-term accumulation of PSR gene transcripts. In this study, the question of whether the shorter term kinetics of Phi suppression were similar to those of P_i_ was addressed (Müller *et al.*, 2004; Morcuende *et al.*, 2007). Organ-level accumulation of both P_i_ and Phi in P-limited seedlings in *A. thaliana* accession Col-0 and three PHT1 transporter mutants was therefore determined. Root growth and anthocyanin accumulation as well as gene expression profiles in response to Phi treatment or P_i_ resupply were monitored in P-limited Col-0 seedlings over a time-course from 1 d to 7 d.

## Materials and methods

### Plant material and growth conditions

Seeds of *A. thaliana* (L.) Heynh. Col-0 and homozygous T-DNA insertion lines for *pht1;1–2* (SALK 088568C) (Shin *et al.*, 2004), *pht1;8* (SALK 056529, Lapis-Gaza *et al.*, 2014), and *pht1;9-1* (SALK 050730) (Remy *et al.*, 2012) were surface-sterilized for 2min in 70% (v/v) ethanol and 5min in 5% (v/v) NaOCl, before being rinsed five times in sterile water. Seeds were resuspended in sterile 0.1% (w/v) agar and stratified in the dark for 24–48h at 4 °C. Seedlings (12 per plate) were grown vertically on 10×10cm plates containing 50ml of nutrient solution [1mM Ca(NO_3_)_2_, 2mM KNO_3_, 0.5mM MgSO_4_, 0.25mM KH_2_PO_4_, 40 μM Fe-EDTA, 25 μM H_3_BO_3_, 2 μM MnCl_2_, 2 μM ZnSO_4_, 0.5 μM CuSO_4_, 0.075 μM (NH_4_)_6_Mo_7_O_24_, 0.15 μM CoCl_2_, 50 μM KCl, pH 5.8] with 0.5% (w/v) 2-(*N*-morpholino)ethanesulphonic acid and 1% (w/v) sucrose, and solidified with 0.7% (w/v) agar (Plant TC Agar, cat.#A111, PhytoTechnology Laboratories, Shawnee Mission, KS, USA). Plates were sealed with 3M™ Micropore medical tape (Intouch Direct, Springwood, Australia). Seedlings were grown in a 10/14h day/night cycle with 200 μmol m^–2^ s^–1^ photosynthetically active radiation (PAR) at 21 °C (day), 19 °C (night), and 65% relative humidity. The plant-available P_i_ present in the agar added another 5 μM to the medium. This amount is within the range of P_i_ concentrations across gelling agents (Jain *et al.*, 2009). Preliminary experiments showed that concentrations of P_i_ ranging from 250 μM to 1mM do not limit seedling growth in this system (data not shown). For the experiment, seedlings grown on a medium with 250 μM P_i_ for 5 d were grown for 4 d on plates without P_i_ supplementation (containing 250 μM KCl instead) before being transferred to plates containing minimal P_i_ (5 μM residual P_i_ in agar), or equimolar concentrations (250 μM) of either P_i_ or Phi. The Phi solution was prepared from a fresh batch of phosphorous acid (99%, Sigma Aldrich, Castle Hill, Australia) as a filter-sterilized 250mM stock. The pH was adjusted to pH 5.8 with KOH. There was <0.1% oxidation of Phi to P_i_ in this solution during 1 month storage at 4 °C.

At harvest, the 12 seedlings on each plate were pooled into one sample. Roots were rinsed in MilliQ water for 5min. Roots and shoots were blotted dry and shock-frozen in liquid nitrogen. Harvesting started 3h after the beginning of the light period in synchrony with the experimental time-course to ensure that plants were at a comparable physiological state.

### Root growth analysis and microscopy

After emergence of the radicle or transfer to a new plate, the position of the primary root tip was marked at 24h intervals. Prior to transfer or harvest, the seedlings were scanned at 600 dpi resolution to determine root and root hair length, growth rate, and lateral root number (LSM Image Browser v4.2; Carl Zeiss Microscopy GmbH, Jena, Germany).

For microscopy (Axioplan Universal microscope; Carl Zeiss Microscopy GmbH), roots were mounted onto slides in water under glass cover slips. Images were electronically processed (AxioVision4; Carl Zeiss Microscopy GmbH).

### Metabolite quantification

Fifteen volumes of 1% (v/v) acetic acid were added to frozen plant powder (30–50mg) and homogenized for three cycles of 45 s at 5000rpm in the presence of two ceramic beads (ø 2mm, Precellys 24 Tissue Disruptor; Bertin Technologies, Montigny-le-Bretonneux, France). After incubation for 15min on ice, the homogenization process was repeated once. Cleared supernatants were used to determine organ P_i_ concentrations via the reduction of a phosphomolybdate complex by ascorbic acid (Ames, 1966). Phi concentrations were determined using the same extracts in a high-throughput enzymatic fluorescence assay (Berkowitz *et al.*, 2011).

Anthocyanins in leaf samples were determined using a pH-differential method (Wrolstad *et al.*, 2005). Concentrations were calculated using the molar absorptivity of cyanidin-3-glucoside (ε=26 900 l mol^–1^ cm^–1^), the predominant anthocyanin in *A. thaliana* leaves (Tohge *et al.*, 2005).

### Relative quantification of transcript abundance

mRNA was captured from tissue homogenates using oligo(dT)_25_-coated magnetic beads (Dynabeads, Life Technologies Australia Pty Ltd, Mulgrave, Australia) and converted to cDNA as previously described (Jost *et al.*, 2007). Aliquots of 0.5ng of cDNA were amplified in a 10 μl reaction volume containing 0.3 μM of each primer and PCR master mix (Power SYBR^®^ Green, Applied Biosystems, Scoresby, Australia). Quantitative PCR and threshold cycle (C_t_) determination were performed using a fluorescence baseline setting of 0.3 (7500 FAST Real-Time PCR System, Applied Biosystems, Scoresby, Australia). Data were normalized against *PP2AA3* (formerly *PDF2*) and *UBC9* reference genes (Czechowski *et al.*, 2005). PCR efficiencies for each primer pair were determined using the LinReg algorithm (Ruijter *et al.*, 2009) (Supplementary Table S1 available at *JXB* online). Data were expressed either relative to normalized C_t_ values in control samples (ΔΔC_t_) or as 40–ΔC_t_ values that correlate with the relative transcript expression of the gene of interest (Bari *et al.*, 2006). The detection limit of the assay was calculated to be a 40–ΔC_t_ value of 25.7±0.1.

### Statistical analysis

Statistically significant differences between treatments were determined using analysis of variance (ANOVA) and defined as *P*≤0.05 (SigmaStat v. 12.3, Systat Software Inc., San Jose, CA, USA). Two-way ANOVA followed by Tukey’s post-hoc test was used to separate means. Hierarchical clustering was performed using squared Euclidean distance and complete linkage [J-Express 2012, Norwegian Bioinformatics Platform and Norwegian Microarray Consortium (http://www.molmine.com)] http://jexpress.bioinfo.no/site/ (last accessed 27 January 2015) (Dysvik and Jonassen, 2001).

## Results

### Phosphite strongly reduced plant biomass production

**Fig. 1.**
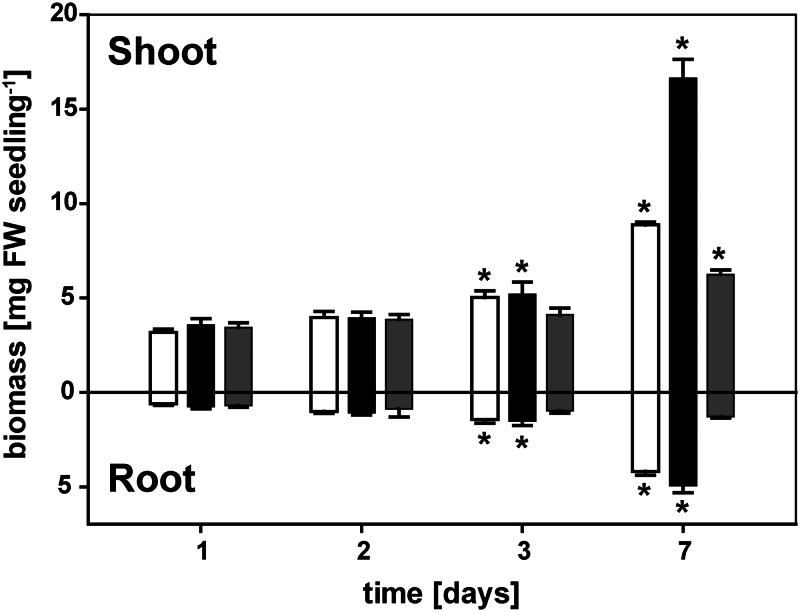
Accumulation of root and shoot biomass. Seeds were germinated on media containing 250 μM phosphate (P_i_) on vertical plates as described in the Materials and methods. Five-day-old seedlings were transferred to a low-P_i_ medium for 4 d before being transferred to plates containing minimal P_i_ (5 μM, white bars), high P_i_ (250 μM, black bars), or phosphite (250 μM, grey bars) media. Root and shoot biomass was determined at 1, 2, 3, and 7 d after transfer (mean ±SE, *n*=4 replicates with 12 seedlings each). Statistically significant differences between time points as determined by Tukey’s HSD for each treatment at *P<*0.001 are indicated by an asterisk. Differences between treatments were significant for both organs only at 7 d after transfer (*P*<0.001).

A vertical growth system was used for *A. thaliana* Col-0 seedlings that allowed a direct comparison of the effects of Phi versus P_i_ on the repression of P_i_-starvation responses without the confounding effects of competition between P_i_ and Phi. Using this system, P-limited seedlings were subjected to continued P_i_ deprivation, P_i_ resupply, or Phi treatment. Plant biomass did not differ significantly among the treatments within the first 2 d of transfer (Fig. 1). After 3 d of treatment, both the P-limited seedlings and those resupplied with P_i_ had greater root and shoot biomass than seedlings at days 1 and 2, while the biomass of the Phi-treated seedlings was unchanged. Over the next 4 d, seedlings resupplied with P_i_ recovered from P limitation with a proportional increase in both root and shoot biomass that maintained the root-to-shoot ratio at 0.30±0.01. P-limited seedlings preferentially allocated resources to roots over shoots, leading to a final root-to-shoot ratio of 0.47±0.03. Despite the greater partitioning of biomass to roots, the root biomass after 7 d of further P limitation was only 84% of that in the P_i_-resupplied seedlings. The shoot biomass of the P-limited seedlings was only 53% of that of the P_i_-resupplied seedlings. By contrast, the Phi treatment slowed seedling growth much more severely. After 7 d exposure to Phi, the final root and shoot biomass of seedlings was only 26% and 38%, respectively, of that in P_i_-resupplied seedlings. While there was a 55% increase in shoot biomass over time, the root biomass of Phi-treated seedlings did not change. Since this severe inhibition of root growth contrasted with both P-limited and P-sufficient seedling growth, the kinetics of root elongation were examined in more detail.

### Phosphite strongly inhibited primary root elongation

**Fig. 2.**
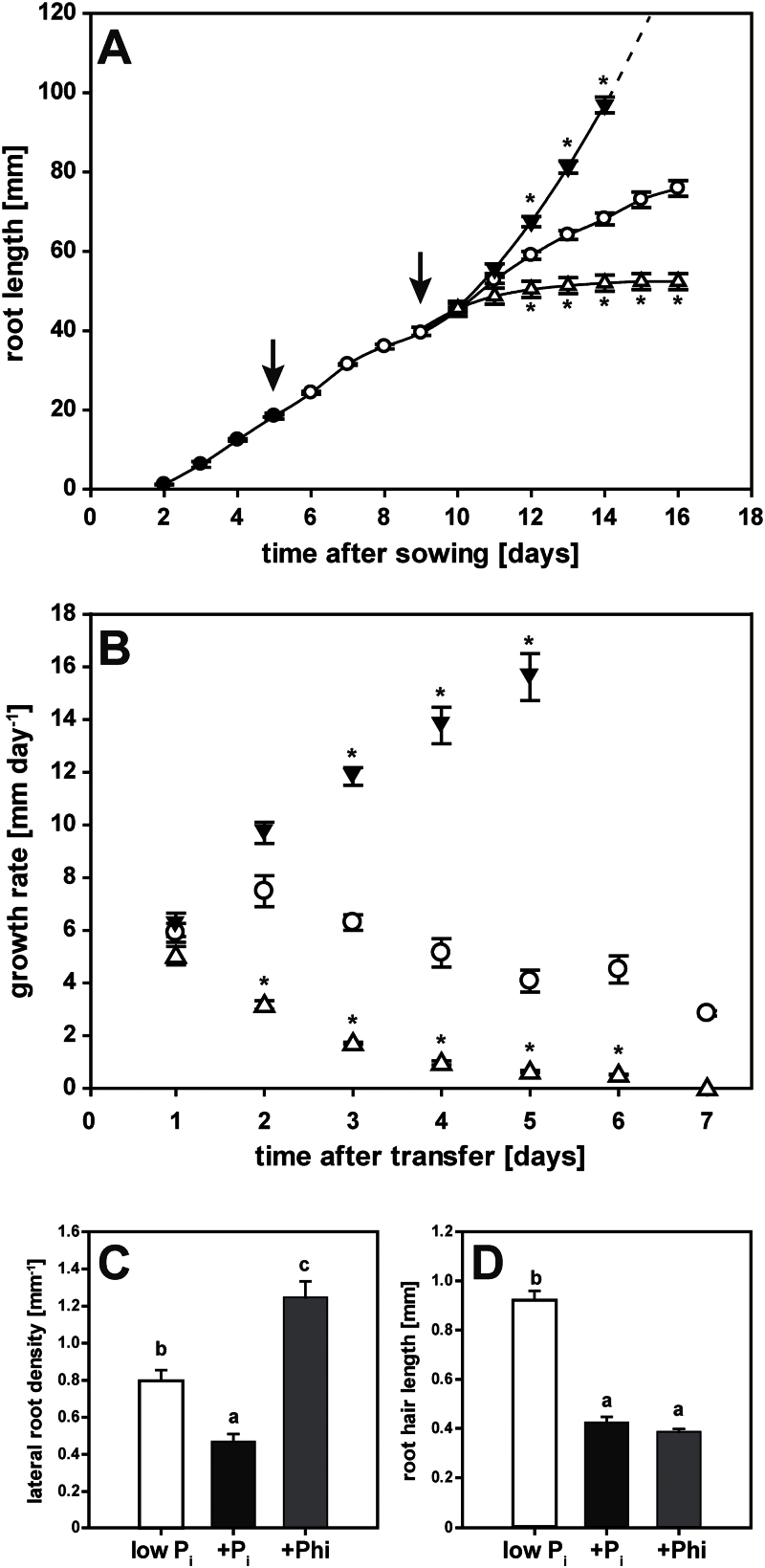
Changes in root architecture in response to phosphate (P_i_) resupply and phosphite (Phi) treatment. (A) Primary root growth over the course of the experiment. Seedlings were germinated on media containing 250 μM P_i_ (filled circles). After 5 d, they were transferred to media containing minimal P_i_ (5 μM, open circles) before being transferred to plates with minimal P_i_ (5 μM, open circles), high P_i_ (250 μM, filled triangles), or Phi (250 μM, open triangles). Arrows indicate transfer to new plates. (B) Root growth rates in response to treatments. Symbols are the same as in (A). Shown in (A, B) are means ±SE, *n*=16 (four seedling roots each were measured individually from four separate plates). (C) Lateral root density in seedlings harvested 7 d after transfer to minimal P_i_ (white bars), high P_i_ (black bars), or 250 μM Phi (grey bars). Emerging lateral roots were counted in root segments that were formed 3 d after transfer. Shown are means ±SE, *n*=10 (five seedlings each from two plates). (D) Root hair length of seedlings harvested 7 d after transfer to minimal P_i_ (white bars), high P_i_ (black bars), or 250 μM Phi (grey bars). Shown are means ±SE, *n*=30 (3 root hairs×5 seedlings×2 plates). Statistically significant differences across time points in (A, B) were determined by Tukey’s HSD for treatments relative to P_i_-limited seedlings at *P*<0.001. In (C) and (D), pairwise multiple comparisons between treatments identified statistically significant differences at *P*<0.005.

Seedlings germinated on high-P_i_ media showed steady primary root growth from 2 d after sowing (Fig. 2A). Root growth was initially maintained when the seedlings were transferred to a P_i_-deficient medium 5 d after sowing. Imposing P_i_ resupply or Phi treatments after 4 d of P_i_ withdrawal did not affect root elongation during the first day (Fig. 2A). Two days after the transfer to the final medium (day 11), roots of both P-limited and P_i_-resupplied seedlings grew at similar rates (Fig. 2B). In contrast, primary roots of Phi-treated seedlings showed much lower growth rates during this period, and elongation ceased completely within the next 48h. Root growth in P_i_-resupplied seedlings accelerated exponentially during this same time period (Fig. 2B), with roots reaching the bottom of the 10-cm plate by 6 d after imposing the treatment. Root growth in P-limited seedlings decelerated by 2%, resulting in a final total root length that was almost 30% shorter than in P_i_-resupplied seedlings. These results show that, unlike P_i_ resupply, Phi treatment accentuated the reduction in root growth caused by P_i_ depletion.

### Phosphite altered seedling root architecture

At the end of the time-course experiment, high-resolution scans of primary root segments initiated on day 3 after the final transfer were used to analyse the effects of the three treatments on root development (Fig. 2C, D). The chosen root segment was proximal to the root apex, at the beginning of the root branching zone (Dubrovsky and Forde, 2012). The short-root phenotype caused by Phi resulted in an almost 2-fold greater lateral root density than in P-limited seedlings in this newly formed section of the root (Fig. 2C). Remarkably, the number of lateral roots per segment in Phi-treated seedlings (2.3±0.2) was 2-fold lower than that in P_i_-limited (4.7±0.3) and P_i_-resupplied (5.0±0.5) segments. Primary root growth in P_i_-resupplied seedlings decreased lateral root density by nearly 2-fold compared with P-limited seedlings. While lateral roots elongated similarly under both P_i_ limitation and P_i_ resupply, emergence of lateral roots was inhibited in the presence of Phi. This phenomenon was also observed in a hydroponics growth system, where transfer to different nutrient solutions is less damaging to roots (Supplementary Fig. S1 at *JXB* online; note that in order to compensate for slower uptake of Phi over P_i_, 1mM Phi was used in this experiment).

Root hairs of Phi-treated seedlings were 57% shorter than in P-limited seedlings (Fig. 2D). This shortening was similar to the 52% reduction observed for seedlings resupplied with P_i_. A concomitant reduction in root hair density by 38% for Phi-treated seedlings (14±1mm^–1^) compared with P-limited seedlings (22±1mm^–1^) was also very similar to the 44% reduction observed in P_i_-resupplied seedlings (13±1mm^–1^). Hence this local response to P_i_ resulting in fewer and shorter root hairs appears to be mimicked by Phi.

### Anthocyanin accumulation in P-limited seedlings was repressed by both P_i_ and Phi

**Fig. 3.**
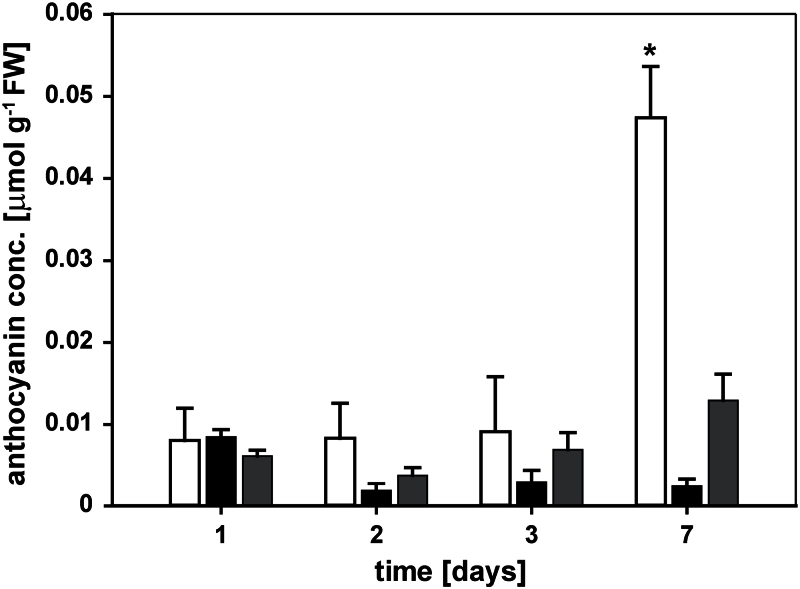
Anthocyanin accumulation in leaves of phosphorus-limited seedlings. Five-day-old seedlings were grown on low-phosphate (P_i_) medium for 4 d before being transferred to plates with minimal P_i_- (5 μM, white bars), high P_i_- (250 μM, black bars), or phosphite- (250 μM, grey bars) containing media. Leaf anthocyanin concentrations were determined at day 1, 2, 3, and 7 after transfer (mean ±SE, *n*=3 replicates with 12 seedlings each). Statistically significant differences between time points and treatments were determined by Tukey’s HSD at *P*<0.001. Differences within the low-P_i_ series and between the low-P_i_ and the other two treatments were significant only at 7 d after transfer (*P*<0.001).

Anthocyanins accumulated to significant levels in leaves of seedlings after a total of 11 d of growth on minimal P_i_ media (4 d P_i_ withdrawal+7 d treatment; Fig. 3). This slow accumulation indicates that the seedlings were not highly stressed by the P_i_ deprivation imposed during the early stage of the experiment, and were probably accessing and gradually depleting P reserves that accumulated during the initial 5-d growth on P_i_-containing medium. Seedlings resupplied with P_i_ after a starvation period of 4 d had lower levels of anthocyanins within 2 d of treatment. Phi-treated seedlings also had reduced leaf anthocyanin levels within the first 2 d of treatment, but not as low as in P_i_-resupplied seedlings. In Phi-treated seedlings, the leaf anthocyanin concentration was higher at day 7 than in P_i_-supplied seedlings, but was 72% lower than in P-limited seedlings. Therefore, Phi attenuated anthocyanin accumulation in P-limited plants that was completely suppressed by P_i_ resupply.

### Root-to-shoot transport favoured P_i_ over Phi

**Fig. 4.**
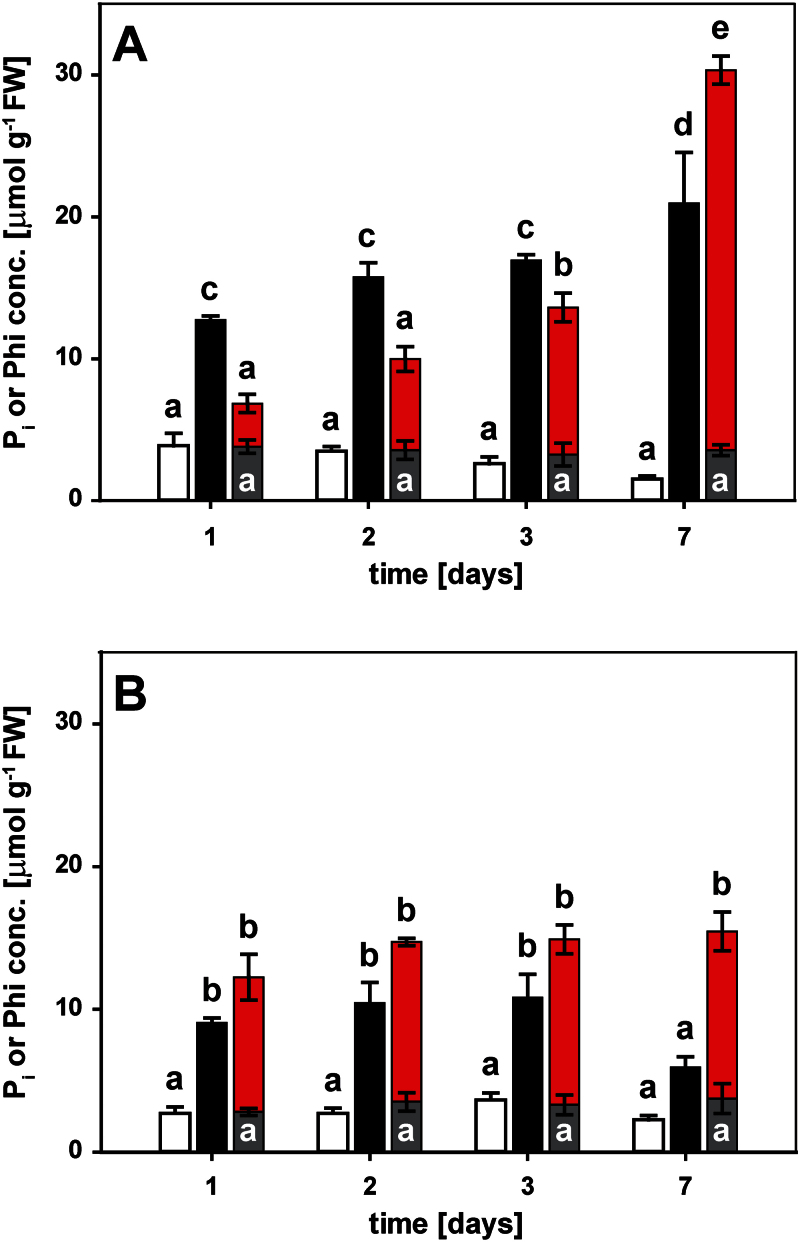
Kinetics of phosphate (P_i_) and phosphite (Phi) accumulation in seedling organs. Five-day-old seedlings were depleted of P_i_ for 4 d before being transferred to plates for the different treatments as indicated. (A) Shoot and (B) root accumulation of P_i_ in phosphorus-limited seedlings (white bars), upon P_i_ resupply (black bars), or with Phi treatment (grey bars). Phi accumulation in Phi-treated seedlings is shown as red bars. Shown are means ±SE, *n*=3 or *n*=4 replicates with 12 seedlings each. Statistically significant differences between time and treatments are indicated by different letters according to Tukey’s HSD at *P*<0.001.

To appreciate fully the differences in the physiological and molecular responses to Phi compared with P_i_, the accumulation of both anions in roots and shoots was determined over time. While roots accumulated both P_i_ and Phi equally within 1 d of exposure, there was a delay in the accumulation of Phi relative to that of P_i_ in the shoot (Fig. 4). Shoot P_i_ concentrations reached ~13 μmol g^–1^ fresh weight (FW) within 1 d of resupply (Fig. 4A) which was greater than the level of free P_i_ in seedlings continuously grown on a sufficient P_i_ supply (~5 μmol g^–1^ FW). The shoot P_i_ concentration nearly doubled over the next 6 d to a final concentration of ~21 μmol g^–1^ FW. In roots, P_i_ levels increased to 9 μmol g^–1^ FW within 1 d of resupply, matching the P_i_ concentration found in roots of seedlings continuously receiving P_i_ (~10 μmol g^–1^ FW). P_i_ concentrations remained at this level for several days, before dropping to 6 μmol g^–1^ FW by day 7 (Fig. 4B). The drop in P_i_ was probably due to a combination of depletion from the medium, continued export to the shoot, conversion to organic P compounds, and internal dilution by root growth. In roots of Phi-treated seedlings, Phi accumulated to similar levels as P_i_ within 1 d, and remained high for the 7 d of the experiment, with a final concentration of 12 μmol g^–1^ FW. In shoots of Phi-treated seedlings, Phi concentrations were lower than the P_i_ concentrations in P_i_-resupplied seedlings at the two earliest time points (3 μmol g^–1^ FW; Fig. 4A). After 3 d, the shoot Phi concentration of 10 μmol g^–1^ FW caught up with the shoot P_i_ concentration found after only 1 d of P_i_ resupply. At the final harvest, the shoot Phi concentration of 27 μmol g^–1^ FW in Phi-treated seedlings was higher than that of the free P_i_ concentration in resupplied seedlings, probably due to metabolic conversion of P_i_ but not Phi into organic compounds. In shoots of P-limited seedlings, the P_i_ concentration tended to decline over the course of the experiment to a final concentration of 1.5 μmol g^–1^ FW. The P_i_ concentration in roots of P-limited plants (2 μmol g^–1^ FW) was constant over the time-course. In roots and shoots of Phi-treated seedlings, the P_i_ concentration (3 μmol and 4 μmol P_i_ g^–1^ FW, respectively) was constant over time, and P_i_ concentrations at the final harvest were higher than those in P-limited organs, most probably due to Phi-induced P_i_ retention in the vacuole (Pratt *et al.*, 2009).

### Phosphite tissue accumulation was differentially affected among a set of *pht1* mutants

**Fig. 5.**
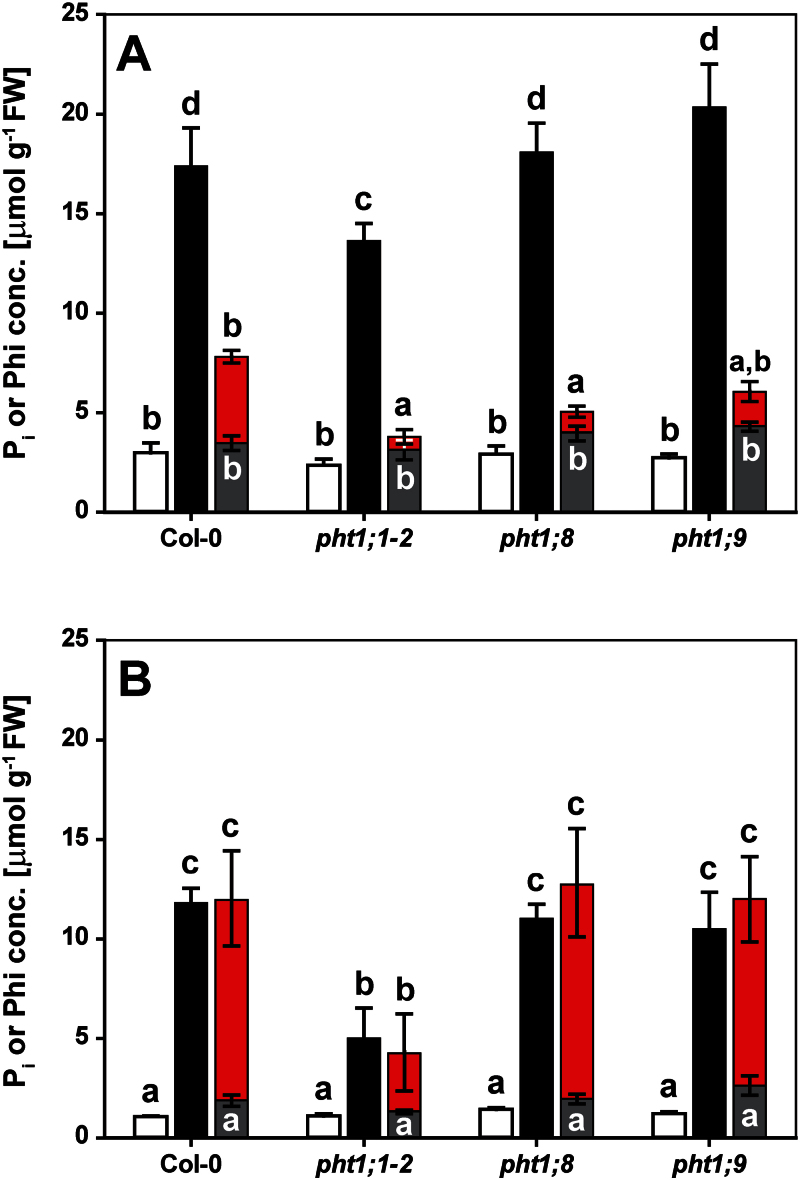
Phosphate (P_i_) and phosphite (Phi) accumulation in P-limited Col-0 and *pht1* mutant organs after 1 d of P_i_ resupply or Phi treatment. Five-day-old seedlings were depleted of P_i_ for 4 d before being treated as indicated. (A) Shoot and (B) root accumulation of P_i_ in P-limited (white bars), P_i_-resupplied (black bars), and Phi-treated (grey bars) seedlings and accumulation of Phi (red bars). Shown are means ±SE, *n*=3 replicates with 12 seedlings each. Genotypes and treatments with a letter in common are not significantly different according to Tukey’s HSD at *P*<0.05.

To gather direct evidence that Phi is transported by P_i_ transporters of the PHT1 family, the Phi accumulation in roots and shoots of homozygous T-DNA insertion lines was analysed in the Col-0 background lacking either PHT1;1, one of the major P_i_ transporters at the root–soil interface (Shin *et al.*, 2004), PHT1;8, or PHT1;9. The latter two PHT1 transporters are involved in translocation of P_i_ to the shoot (Lapis-Gaza *et al.*, 2014). Seedlings were grown on vertical plates and depleted of P_i_ as described above, before supplying them with either 250 μM P_i_ or 250 μM Phi for 24h prior to harvest. P_i_ starvation led to similar residual organ P_i_ concentrations across genotypes (Fig. 5). Compared with the corresponding wild-type Col-0, the *pht1;1–2* mutant accumulated 58% less P_i_ in roots and 22% less P_i_ in shoots of P_i_-resupplied seedlings over the 24-h period (Fig. 5). The effect of this mutation on Phi uptake by P-limited seedlings was significantly more pronounced, leading to 71% less Phi in roots and 84% less Phi in shoots of the mutant than in the wild type. Knocking out *PHT1;8* or *PHT1;9* had no effect on either root or shoot P_i_ accumulation. In contrast to *pht1;1–2*, Phi concentrations in roots of both *pht1;8* and *pht1;9-1* were similar to those in the wild type, but Phi accumulation in shoots was reduced by 76% for *pht1;8* and by 60% for *pht1;9-1* compared with Col-0, the same extent as seen in *pht1;1–2*. The basal organ P_i_ concentrations in Phi-treated seedlings were similar across mutants. The same trends in organ P_i_ and Phi concentrations were observed after 2 d of treatment, although differences between Col-0 and the three mutants were diminished by day 7 (Supplementary Fig. S2 at *JXB* online). Throughout the time-course, root and shoot biomass accumulation was largely unaffected by the lack of individual PHT1 proteins (Supplementary Fig. S3).

### Phosphite altered transcript accumulation for a subset of P_i_-responsive genes

The short-term effect of Phi on PSR gene expression was assessed by quantitative reverse-transcriptase PCR (qRT-PCR) for a set of well-documented PSR genes representing various metabolic and regulatory steps within plant P signalling networks (Hammond *et al.*, 2003; Wu *et al.*, 2003; Misson *et al.*, 2005; Morcuende *et al.*, 2007; Woo *et al.*, 2012). If Phi was a true P_i_ analogue and sensed in the same way as P_i_ by as yet unidentified cellular signalling components, one would expect the effect of the two chemicals on transcript profiles to be similar; that is, lower transcript levels for P_i_-starvation-induced genes and higher transcript abundance for genes involved in organophosphate biosynthesis or encoding negative regulators such as the E2 ubiquitin conjugase PHO2 (Aung *et al.*, 2006; Bari *et al.*, 2006) or the F-box protein FBX2 and transcription factor BHLH32 (Chen *et al.*, 2008).

**Fig. 6.**
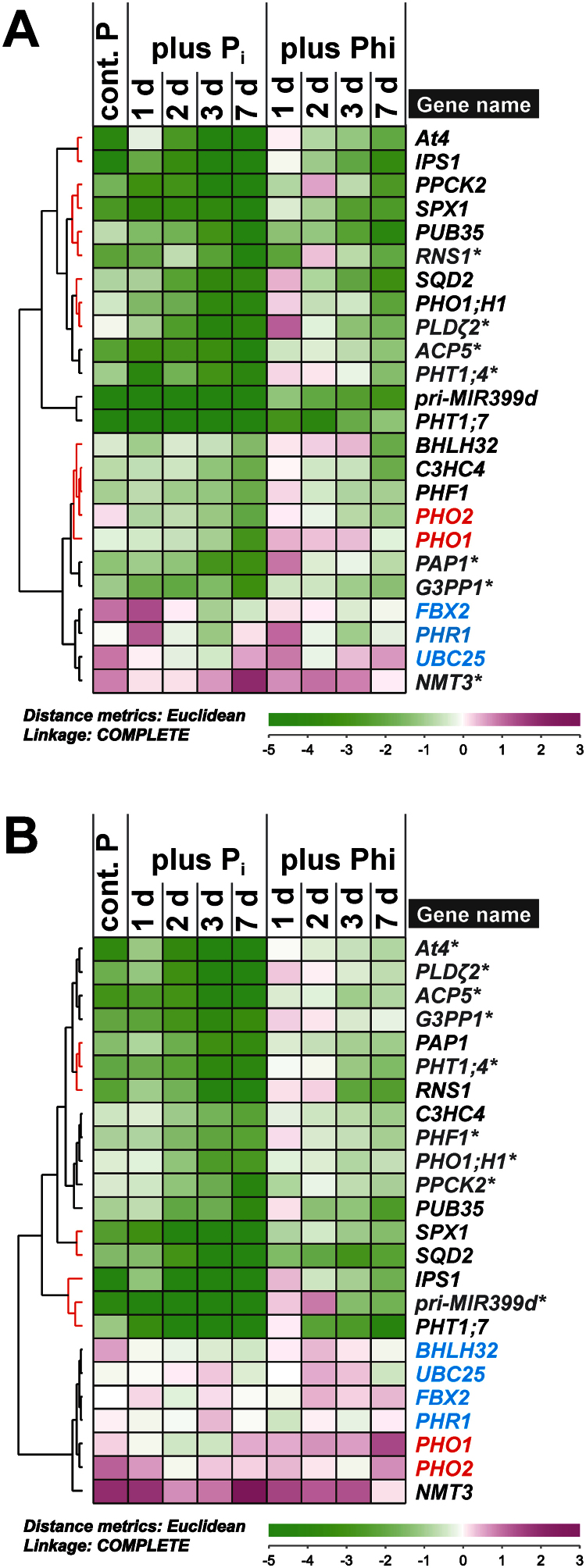
Effect of phosphate (P_i_) and phosphite (Phi) on transcript abundance in P-limited seedlings. Hierarchical cluster analysis of a time-course on relative transcript abundance in P-limited *Arabidopsis thaliana* (A) shoots and (B) roots in response to P_i_ resupply or Phi treatment. Mean log2 expression ratios (–ΔΔC_t_) relative to the normalized expression in P-limited plants with three biological replicates for each sample are shown. Raw data were normalized against the transcript abundance of *PP2AA3* and *UBC9* reference genes. Clusters that contain Phi-responsive transcripts are highlighted by red lines in the tree. Transcripts in black change abundance in response to both Pi and Phi treatment, while those in grey (*) are unresponsive to Phi treatment (*P*≤0.05). *PHO1* and *PHO2* transcripts are highlighted in red. Transcripts in blue show no significant change in abundance across treatments. Details on individual transcript expression patterns and statistical analysis can be found in Supplementary Table S2 at *JXB* online.

The selected PSR genes showed the previously documented expression changes within 1 d of P_i_ resupply (Fig. 6). Surprisingly, 33% of the target genes showed no significant change in transcript abundance in response to Phi in shoots of P-limited plants over the 7-d treatment period (Fig. 6A, grey transcript names). Within this non-responsive group were the P_i_ transporter gene *PHT1;4*, as well as genes involved in P_i_ metabolism (*ACP5*, *G3PP1*, *NMT3*, *PAP1*, *PLD ζ2*, and *RNS1*). In shoots, the majority of PSR genes tested showed an attenuated response to Phi treatment with a 1 d or 2 d delay compared with P_i_ resupply. This set included genes encoding regulatory components such as At4, IPS1, PHO1;H1, and SPX1, as well as genes encoding protein kinase PPCK2 and sulpholipid synthase SQD2 (Fig. 6A, red clusters). In contrast, other genes responded strongly to Phi, as they did to P_i_ resupply. These responses included an 8-fold suppression within 24h of Phi treatment for *PHT1;7* transcript amounts, with a further 16-fold drop within 2 d of treatment. Similarly, transcripts encoding U-box-containing E3 ligase PUB35 were less abundant in shoots within 24h of Phi treatment. A milder suppression compared with P_i_ was observed for the primary transcript of regulatory microRNA miR399d. Transcripts encoding transcription factor BHLH32, E3 ubiquitin ligase C3HC4, and transport facilitator PHF1 responded more slowly but similarly to both P_i_ resupply and Phi treatment, with a >4-fold lower abundance than in shoots of P-limited seedlings at the end of the experiment. *PHO2* transcripts showed an unexpected profile in shoots, with 2- to 4-fold lower levels in P_i_-resupplied over P-limited seedlings. Phi treatment triggered a similar 2-fold decline in *PHO2* transcripts within 3 d of treatment.

Despite the fact that Phi accumulated as quickly as P_i_ in roots, 43% of the tested P-responsive transcripts did not respond to Phi in this organ (Fig. 6B, grey transcript names). Transcripts from *ACP5*, *G3PP1*, *PLDζ2*, and *PHT1;4* were among those that were also identified as being non-responsive to Phi in shoots. In roots, Phi-non-responsive transcripts included those from *At4*, *PHO1;H1*, *PHF1*, and *PPCK2*, all of which responded to Phi to some extent in shoots. On the other hand, transcripts encoding phosphatase PAP1 and ribonuclease RNS1 were more responsive to Phi in roots compared with shoots. As in shoots, *NMT3* transcript abundance in roots increased 2-fold in response to Phi within 24h of treatment, but transcript levels did not continue to increase and were 11-fold lower compared with roots of P_i_-resupplied plants on day 7 (Supplementaty Table S2 at *JXB* online). *PHT1;7*, *PUB35*, *SPX1*, and *SQD2* were highly Phi responsive in roots as well as in shoots. However, the response was relatively delayed in roots for *PHT1;7* and *PUB35*, while *SPX1* and *SQD2* transcripts were more quickly suppressed in roots than in shoots. *C3HC4*, *IPS1*, and *pri-MIR399d* transcript abundance showed a weaker response to Phi in roots compared with shoots. In contrast to shoots, *PHO1* transcript abundance did not respond to P_i_ resupply in roots. Curiously, within 48h of Phi treatment, *PHO1* transcript abundance was ~2-fold greater than that in roots of P-limited plants and continued to increase throughout the time-course. *PHO2* transcript abundance in roots did not respond to either P_i_ or Phi treatment.

It has to be noted that seedlings were not severely P starved at the beginning of the experiment. Evidence for this was the small changes in transcript abundance in organs of P-limited control plants at day 1 of the experiment compared with transcript levels in plants continuously supplied with P_i_ (blue bar in Supplementary Table S2 at *JXB* online). As a consequence, transcript levels of the target P_i_-starvation-induced genes continued to increase over the time-course in P-limited control plants. This was also the case for those transcripts that did not show a response to Phi in Phi-treated seedlings.

In contrast to the gradual response to P_i_ deprivation, P_i_ resupply led to the suppression of P_i_-starvation-induced genes within 24h (Supplementary Table S2 at *JXB* online). Thereafter, transcript abundance remained at the newly established lower levels for the rest of the time-course. Exceptions to this expression profile were those of microRNA antagonists *IPS1* and *At4*, which showed a more gradual response to P_i_ resupply in both roots and shoots. In shoots, *PHT1;4* transcripts also showed this gradual decrease in abundance in response to P_i_. Unlike all other target genes, transcripts from both *IPS1* and *PHT1;4* decreased in abundance to below the level observed in shoots of seedlings that were continuously supplied with P_i_. In roots, *PHO2* transcript levels tended to increase transiently within 24h of P_i_ resupply, rather than showing a sustained increase over P-limited plants. *PHO2* transcripts did not respond to P_i_ resupply in shoots.

## Discussion

Phi has been demonstrated to suppress the induction of P_i_-starvation responses. This conclusion was drawn from a series of experiments where P-sufficient plants were transferred to P_i_-containing or P_i_-free media supplemented with increasing Phi concentrations, or where seeds were germinated on these media (Carswell *et al.*, 1996; Ticconi *et al.*, 2001; Varadarajan *et al.*, 2002; Berkowitz *et al.*, 2013; Eshraghi *et al.*, 2014). Thus, these studies focused on the ability of Phi to interfere with the induction of PSR genes in response to P_i_ removal or the lack of P_i_ supply. The experimental set-up used in this study allowed direct comparison of Phi and P_i_ effects on the suppression of P_i_-starvation responses through monitoring plant growth, P_i_ anion and anthocyanin accumulation, as well as PSR gene expression. The experimental set-up has several advantages. (i) Withdrawal of P_i_ from the medium prior to Phi treatment avoids competition between the two anions for uptake. (ii) A direct comparison of Phi and P_i_ effects on the suppression of PSR genes can be conducted. (iii) Phi accumulation in the cytosol and organelles should be favoured over the vacuole under these conditions, so that more direct effects on metabolism and gene regulatory networks can be observed. (iv) The kinetic dependences of these effects on the accumulation of both P anions in roots and shoots can be determined.

### Discrimination between P_i_ and Phi by PHT1 transporters

The differential movement of Phi and P_i_ into the shoots of plants suggests different affinities for these molecules within their transport routes. Measurements of transport kinetics in different systems have concluded that P_i_ transporters are able to transport Phi, albeit with a lower affinity than for P_i_ (d’arcy-Lameta and Bompeix, 1991; Pratt *et al.*, 2004; Danova-Alt *et al.*, 2008; Basheer *et al.*, 2011). This means that Phi can bind to P_i_ transporter proteins without inducing the same conformational changes necessary for efficient transport (Basheer *et al.*, 2011). It is unknown if all plant PHT transporters interact with Phi with the same affinity or whether some discriminate more strongly against Phi. In this study, the more pronounced delay in root-to-shoot transport of Phi in the *pht1;8* and *pht1;9-1* mutants than in wild-type seedlings, without a delay in Phi uptake, suggests that the encoded transporters discriminate more strongly against Phi than PHT1;1. The fact that discrimination is stronger in the absence of either PHT1;8 or PHT1;9 could mean that the two only partially complement each other (Lapis-Gaza *et al.*, 2014) which would slow down transport even further. Alternatively, a third transport process, perhaps involving the P_i_ exporter PHO1 (Arpat *et al.*, 2012), could be implicated in the stronger discrimination between P_i_ and Phi in both mutants. The alleviation of the Phi discrimination phenotype over time is most probably due to remobilization processes between sink and source organs involving other PHT transporters, such as PHT1;5 (Nagarajan *et al.*, 2011).

Differential recognition of Phi by different PHT proteins may modulate not only transport activity, but also signalling events associated with this activity (Schothorst *et al.*, 2013). It is unclear whether such a ‘transceptor’ function applies to the plant PHT family, but complex post-translational regulation has already been shown. Bayle *et al.* (2011) showed that some high-affinity PHT1 proteins undergo complex post-translational modifications, including protein phosphorylation. PHT1 protein abundance is also controlled by ubiquitin-mediated protein degradation (Lin *et al.*, 2013; Park *et al.*, 2014). Both PHT1;8 and PHT1;9 proteins can be distinguished from other family members by the presence of a PEST [*p*roline, glutamic acid (*E*), *s*erine, *t*hreonine] domain that mediates phosphorylation-dependent protein degradation in many systems (Rechsteiner and Rogers, 1996), for example the high- and low-affinity P_i_ transporters in yeast (Lagerstedt *et al.*, 2004; Estrella *et al.*, 2008).

### Differential expression of ‘PHO regulon’ genes in response to local P_i_ signalling in roots and shoots

**Fig. 7.**
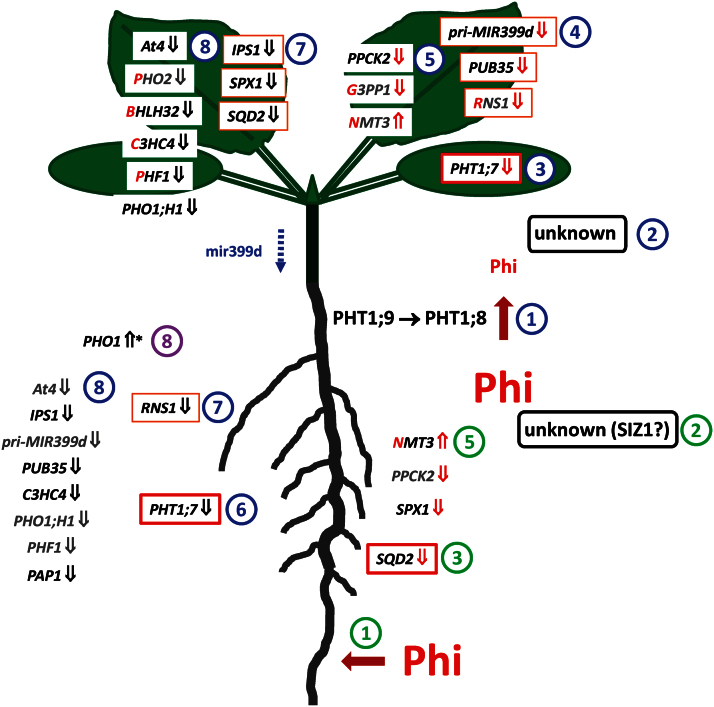
A model for the sequence of changes in phosphate-starvation-responsive (PSR) gene expression observed in roots and shoots of phosphorus-limited *Arabidopsis thaliana* seedlings in response to phosphite (Phi) treatment. Blue pathway: the discrimination of Phi by PHT1;9 and subsequently by PHT1;8 during xylem loading (1) may indicate the recognition by a receptor that signals the availability of phosphate (P_i_) and Phi to the shoot, possibly involving SIZ1 (2). This sequence of events may primarily affect *PHT1;7* expression in the shoot (3), followed by the consecutive suppression of other PSR genes within 24h (4+5) or later after 3 d when Phi finally started to accumulate in the shoot (7+8). Green pathway: in roots, early local recognition of Phi is possibly restricted to the suppression of *SQD2* within 24h (3) and of the less responsive *SPX1* and *PPCK2* as well as to the induction of *NMT3* (5). Compared with shoots, *PHT1;7*, *RNS1* and a couple of transcripts in group 8 (in grey) responded more slowly in roots, most probably indicating that their expression in roots is regulated by PHO2 and relies on systemic signalling, perhaps through reduced levels of mir399d in the phloem (blue dotted arrow). Curiously, *PHO1* expression in roots increased within 3 d of Phi treatment, which may indicate its connection to independent regulatory networks (purple 8) that directly respond to the overall P status of the plant or the growth inhibition triggered by Phi. Note that the number of genes responding equally well to either P_i_ or Phi (red first letter in gene name) was greater in shoots than in roots. Gene names in black indicate a 2-fold expression change in response to Phi over P-limited controls (Fig. 6). An orange border indicates a 4-fold expression change. A bold red border indicates an 8-fold change. Grey names indicate non-significant changes. Red arrows following gene names indicate suppression (↓) or induction (↑) within 24h of Phi exposure, while black arrows indicate a response within 3 d of treatment. An asterisk indicates a Phi-specific expression change that was not observed in P_i_-resupplied seedlings.

There is mounting evidence that the local and systemic control of PSR gene expression is governed by different signalling circuits in roots and shoots, and that different circuits within each organ respond either to the direct perception of P_i_ or to a more indirect process involving downstream metabolites or other as yet unidentified signals (Müller *et al.*, 2004; Bari *et al.*, 2006; Thibaud *et al.*, 2010; Woo *et al.*, 2012; Rojas-Triana *et al.*, 2013). The discrimination between P_i_ and Phi by PHT1;8 and PHT1;9 shown in this study leads to a delayed accumulation of Phi in shoots. This delayed accumulation of Phi may hence be an elegant tool for dissecting direct sensing of P_i_ from other potential signals of P status in the shoot. *PHT1;7* and *pri-MIR399d* transcripts in the present study were suppressed earlier in shoots than in roots and responded before Phi accumulated to significant levels. This would place them into an early-response circuit more directly connected to a P_i_-specific sensor in the root-to-shoot transport route. *PHT1;7* and *pri-MIR399d* expression was deregulated in the *pht1;9-1* (Lapis-Gaza *et al.*, 2014) and the *phr1* mutant, but not the *pho2* mutant (Bari *et al.*, 2006). Slower shoot accumulation of Phi correlated with an attenuated down-regulation of a select subset of PSR genes closely associated with the ‘PHO regulon’, such as *At4*, *IPS1*, *SPX1*, *PHF1*, and *PHO1;H1*. This would support their response to local P_i_ or, in this case, Phi availability in the shoot. Interestingly, these genes were also deregulated in both P-limited *phr1* and P_i_-resupplied *pho2* mutants (Bari *et al.*, 2006). These findings may indicate that early P_i_- and Phi-responsive genes are more directly connected to PHR1, possibly through a SIZ1-co-ordinated network in roots (Miura *et al.*, 2005) and an unknown signalling component in shoots (Fig. 7) (Klecker *et al.*, 2014). Only very few locally responsive PSR genes in the shoot seem to be PHO2 dependent (Pant *et al.*, 2015). In a split-root system, genes that were systemically regulated in roots showed a strong enrichment of the P1BS element for PHR1 binding in their promoter regions (Thibaud *et al.*, 2010). This may indicate differences in signal perception between roots and shoots. A clear distinction of regulatory groups of genes according to their responsiveness to P_i_ and Phi in space and time would therefore be useful to define individual response circuits further.

*PHO1* transcripts encoding a Golgi-localized P_i_ exporter (Arpat *et al.*, 2012) showed a contrasting expression profile in roots to that of the other PSR genes tested in this study: instead of being suppressed by either P_i_ or Phi addition, they were more abundant in roots of Phi-treated compared with P-limited plants and did not respond to P_i_ resupply. In shoots, P_i_ resupply caused the down-regulation of *PHO1*, while Phi treatment caused a transient increase in *PHO1* transcript levels similar to its effect in roots. *PHO1* is therefore the only PSR gene tested that responded to the more severe depletion of local cytosolic P_i_ pools that is expected in the presence of Phi (Pratt *et al.*, 2009). Alternatively, *PHO1* expression may be triggered by the strong inhibition of seedling growth in the presence of Phi. In this context, it is interesting to note that shoot growth in transgenic lines with reduced *PHO1* expression is uncoupled from the actual P status of the shoot (Rouached *et al.*, 2011). PHO1-associated signalling components could therefore integrate growth stimuli and P status.

*PHO1;H1* transcript accumulation was suppressed by P_i_ in both roots and shoots, with a strong suppression by Phi in shoots. These results confirm the findings of Stefanovic *et al.* (2007) showing that PHR1-dependent *PHO1;H1* expression is Phi responsive, while PHR1-independent *PHO1* expression is not. PHO1 and PHO1;H1 are SPX (*S*YG1, *P*ho81, and *X*PR1) domain proteins (Secco *et al.*, 2012). Transcripts encoding another SPX domain protein, SPX1, responded to Phi in both roots and shoots. SPX1 is a competitive inhibitor of PHR1 binding to the P1BS element in PSR gene promoters (Puga *et al.*, 2014). Its interaction with PHR1 is also highly dependent on the presence of either P_i_ or Phi. In contrast to most PSR genes in the present study, it responded much more quickly to Phi in roots. Both *PHO1;H1* and *SPX1* are regulated in a PHR1- and PHO2-dependent manner (Bari *et al.*, 2006), but *SPX1* is also controlled by SIZ1 (Duan *et al.*, 2008). The latter may explain its more direct response to local Phi concentrations in the root (Miura *et al.*, 2011). This would put SIZ1 into a position close to the local P_i_- and Phi-sensing module in roots (Fig. 7). Surprisingly, SPX1 is also systemically regulated in P-limited roots in a split-root system (Thibaud *et al.*, 2010).

*PHO2* transcripts encoding an E2 ubiquitin conjugase (Aung *et al.*, 2006; Bari *et al.*, 2006) accumulated transiently in P_i_-resupplied roots, but were largely unresponsive to Phi treatment. This suggests that *PHO2* is connected to a signalling circuit that responds very sensitively to changes in overall P status, perhaps through monitoring concentrations of a downstream P metabolite (Klecker *et al.*, 2014; Pant *et al.*, 2015). In support of this interpretation, P-sufficient *pht1;9* mutants showed a stronger accumulation of *At4* and *pri-MIR399d* transcripts, and lower transcript accumulation of *PHO2* in shoots which did not correlate with P_i_ concentrations in *pht1;9* roots or shoots (Lapis-Gaza *et al.*, 2014). The decline in *PHO2* transcripts over the treatment period could therefore be an early response to the P_i_ depletion of the media resulting in lower levels of a downstream P metabolite. This P_i_ depletion after 7 d of treatment would also explain the observed lower P_i_ concentration and the higher transcript abundance for *PHO1* and *SPX1* in roots as well as increasing transcript levels for *PHT1;7* and *pri-MIR399d* in shoots of P_i_-resupplied seedlings. In shoots of P_i_-resupplied seedlings, *PHO2* expression was even lower than that in P-limited seedlings. Since *At4* and *IPS1* transcript levels were significantly lower in shoots in response to either P_i_ or Phi treatment, the late increase in *pri-MIR399d* transcript abundance, which underlies *PHO2* repression, might explain the further drop in *PHO2* transcript amounts in the shoot. In contrast to roots, this response was mimicked by Phi to some extent, again highlighting the differences in P_i_ perception between the two organs.

All the genes mentioned in this section respond very quickly to changes in P status. However, there is a clear distinction in the regulation of *SPX1* that responds very early in roots, *PHO1*, which seems to respond to signals associated with growth, *PHO2* which responds to unknown downstream P signals, and all other components of the ‘PHO regulon’ that do show strong responses to both P_i_ and Phi, especially in shoots. It is possible that the first perception of P_i_ takes place during root-to-shoot transport or within the shoot itself. Conversely, PSR gene expression in the root largely responds to secondary, shoot-derived signals as previously demonstrated (Bari *et al.*, 2006; Lin *et al.*, 2008; Thibaud *et al.*, 2010).

### Phosphite-dependent expression changes in roots affect transcripts for local lipid-remodelling pathways

In roots, transcripts encoding sulpholipid synthase SQD2 that catalyses the last step in sulpholipid biosynthesis and phosphoethanolamine *N*-methyltransferase NMT3 that synthesizes the head group of the phospholipid phosphatidylcholine responded very quickly to both P_i_ and Phi, while their response was slower in shoots. By contrast, *PLDζ2* transcripts encoding a phospholipase D isoform showed a response to P_i_, but not to Phi. *NMT3* is one of the few genes that respond to P_i_ independently of PHR1 and PHO2 in *A. thaliana* seedlings (Bari *et al.*, 2006). In the study of Woo *et al.* (2012), many lipid-remodelling genes such as *PLDζ2* and *SQD2* were among the group of genes that specifically responded to P_i_ in both roots and shoots. Their response to P_i_ was PHR1-dependent, but undisturbed in *pho2* seedlings (Bari *et al.*, 2006). They were also systemically regulated in a split-root system, but their induction was attenuated compared with that in P-limited control roots (Thibaud *et al.*, 2010). These genes were also highly responsive to Phi in an earlier acclimation study (Berkowitz *et al.*, 2013). Kobayashi *et al.* (2006) demonstrated that the promoter of another lipid-remodelling gene, *MGD2*, responds very strongly to Phi in roots and shoots, and that Phi is able to cause the modification of shoot lipid profiles in a similar fashion to P_i_, including lower proportions of galactolipids and sulpholipids, and higher proportions of phospholipids. These findings indicate that direct sensing of P_i_ affects the lipid-remodelling pathway through PHR1-dependent and PHR1-independent signalling cascades, with a more rapid local perception of P_i_ in roots.

### Phosphite effects on root architecture may be caused by altered accumulation of transcripts encoding proteins involved in protein turnover and vesicle trafficking

In this study, a U-box/ARM-repeat E3 ligase gene, *PUB35*, was responsive to the plant’s P status and was among a small group of PSR genes that were highly responsive to Phi, especially in roots. U-box and RING-finger E3 ligases, such as PUB35 and C3HC4, that responded to both P_i_ and Phi in the present study, are highly responsive to the plant’s P status, with many of them showing PHR1/PHL1-dependent regulation (Rojas-Triana *et al.*, 2013). The U-box E3 ligase, PUB9, has recently been implicated in linking auxin-dependent and P_i_-regulated lateral root emergence with vesicle trafficking. PUB9 interacts with the S-domain receptor kinase ARK2 that is implicated in P-derived signal recognition (Deb *et al.*, 2014). The *ark2-1*/*pub9-1* double mutant features shorter primary roots under low P_i_ supply, thus mimicking the Phi-induced phenotype in this study. While it has been demonstrated that several PUB E3 ligases can interact with ARK2 *in vitro* (Samuel *et al.*, 2008), this has yet to be demonstrated for PUB35.

What makes this potential link between P_i_ signalling and Phi recognition particularly intriguing is the fact that U-box proteins have also been implicated in triggering plant immunity (Gonzalez-Lamothe *et al.*, 2006; Trujillo *et al.*, 2008). Many of the 64 predicted U-box-containing proteins in *A. thaliana* are associated with mono-ubiquitination and proteasomal degradation of signalling components during stress responses that trigger cell death (Yee and Goring, 2009).

### Indirect effects of Phi treatment on plant growth

In P-limited cell suspension cultures, Phi exacerbates P_i_ starvation by inhibiting vacuolar efflux of P_i_ (Pratt *et al.*, 2009). This could explain the arrest in primary and lateral root growth observed upon Phi treatment in this study and upon longer term Phi exposure (Berkowitz *et al.*, 2013; Eshraghi *et al.*, 2014). In both instances, the growth arrest was much more severe than the slowing of primary root elongation observed upon P_i_ withdrawal alone. However, plants in the present study were not experiencing severe P_i_ starvation, given that anthocyanin levels in leaves of P-limited controls only started to increase towards the end of the experiment. Also, a significant reduction of both root and shoot biomass in the presence of Phi compared with that of both continued P-limited growth and P_i_ resupply was only observed on the third day of treatment, at the time when Phi first significantly accumulated in shoots. The inhibitory effect that Phi has on organ growth might thus be directly triggered by the accumulation of Phi within the cytosol and organelles of the shoot (Danova-Alt *et al.*, 2008; Pratt *et al.*, 2009). As would be expected from a mildly cytotoxic agent, Phi then seems to affect both root and shoot growth in a similar fashion. This is very different from the opposing hormonal effects on root and leaf development (King *et al.*, 1995; Werner *et al.*, 2003).

These observations would imply that whilst Phi is a great tool to tease apart direct, P_i_-triggered effects on P signalling networks from those further downstream, care has to be taken to interpret longer term effects due to its immediate toxicity on many P_i_-dependent metabolic pathways.

## Conclusion

The present results indicate that Phi is perceived as P_i_ and suggest that this perception is stronger in shoots than in roots. The perception of Phi most probably affects distinct regulatory circuits in both organs, and is more closely associated with factors that interact with PHR1-associated networks, such as SIZ1 (Miura *et al.*, 2011). The strong root architectural changes induced by Phi in P-limited plants are most probably invoked by its interference with local signalling components that affect lipid remodelling (*PLDζ2*, *SQD2*, *NMT3*) and protein turnover (*PUB35*, *C3HC4*). In the longer term, Phi severely affects plant growth, most probably by inhibiting vital P_i_-dependent metabolic pathways. Several of these pathways have the potential to trigger the priming of plant defences. Used with caution, Phi can be a useful tool in further disentangling these complex interactions.

## Supplementary data

Supplementary data are available at *JXB* online.

Figure S1. Root phenotypic responses in phosphorus-limited plants to phosphate resupply or phosphite treatment.

Figure S2. Phosphate and phosphite accumulation in roots and shoots of Col-0 and *pht1* mutants over time.

Figure S3. Biomass accumulation in roots and shoots of Col-0 and *pht1* mutants over time.

Table S1. Information on target genes and primers used in qRT-PCR analyses.

Table S2. Time-course of relative transcript abundance of known phosphate-responsive genes in phosphorus-limited *A. thaliana* seedlings in response to either phosphate resupply or phosphite treatment.

Supplementary Data

## Abbreviations:

P: phosphorus
Phi: phosphite
P_i_: inorganic phosphorus/phosphate/H_2_PO_4_–
PSR: phosphate-starvation-responsive.
