# Supplementary material for: Differentiating phosphate-dependent and phosphate-independent systemic phosphate-starvation response networks in Arabidopsis thaliana through the application of phosphite
Source: J Exp Bot. 2015 Feb 19;66(9):2501–14. doi: 10.1093/jxb/erv025 (PMC4986860; doi:10.1093/jxb/erv025)
Supplement: Supplementary Data [file supp_66_9_2501__index.html]

Differentiating phosphate-dependent and phosphate-independent systemic phosphate-starvation response networks in Arabidopsis thaliana through the application of phosphite — Differentiating phosphate-dependent and phosphate-independent systemic phosphate-starvation response networks in Arabidopsis thaliana through the application of phosphite — Supplementary Data 

# Differentiating phosphate-dependent and phosphate-independent systemic phosphate-starvation response networks in *Arabidopsis thaliana* through the application of phosphite

## Supplementary Data

Data files

**Files in this Data Supplement:**

- Supplementary Data - Supplementary Data
