# Supplementary material for: Differentiating phosphate-dependent and phosphate-independent systemic phosphate-starvation response networks in Arabidopsis thaliana through the application of phosphite
Source: J Exp Bot. 2015 Feb 19;66(9):2501–14. doi: 10.1093/jxb/erv025 (PMC4986860; doi:10.1093/jxb/erv025)
Supplement: Supplementary Data [file supp_erv025_jexbot141010_file001.pdf]

Supplemental Data

Supplemental Table S1: Information on target genes and primers used in qRT-PCR analyses.

legend:

all primers were designed in Primer Express, spanning exon-intron borders whereever possible and were subsequently checked in Primer-BLAST for sequence specificity

* PCR efficiencies of primer pairs were calculated across all samples using LinReg v. 11.0 (Ramakers *et al.* 2003, Ruijter *et al.* 2009), outliers differing from the means by more than 15 % were excluded

GG = exon/exon junction

gene-specific primer sequence

name	AGI	annotation / alternative name	reference	forward primer (5' → 3')	Tm	reverse primer (5' → 3')	Tm	exon-intron junction?	product (cDNA) [bp]	product (gDNA)	PCR efficiency*
**reference genes**											
PP2AA3	At1g13320	PROTEIN PHOSPHATASE 2A SUBUNIT A3	Czechowski *et al.* (2005)	GCAGCATATAGTTCCTCAGGTTCTAGA	61.9	TTGGCCACGTTAAATTTGATGTT	58.1	3rd last intron	189	none	1.85 ± 0.01
UBC9	At4g27960	UBIQUITIN CONJUGATING ENZYME 9	Czechowski *et al.* (2005)	CCATTGGATTGGTTTTCGATTGCAGA	62.7	GCAACGGGTCCTGCGCTACA	65.5	3rd last intron	142	none	1.82 ± 0.05
**P signaling genes (published)**											
ACP5	At3g17790	ACID PHOSPHATASE type 5, PURPLE ACID PHOSPHATASE 17, PAP17	del Pozo *et al.* (1999)	GGAGATATTAACCCAGTGACCATCA	59.9	AACCGAGCAGACATGAATCCTT	60.0	intron too far away from 3' UTR	80	same as cDNA	1.82 ± 0.02
At4	At5g03545	AT4, ATIPS2, INDUCED BY PI STARVATION 2	Shin *et al.* (2006)	GAGCGATGAAGATTGCATGAAG	58.1	GATCGAAGTTGCCCAAACGA	58.9	no intron	80	same as cDNA	1.88 ± 0.01
BHLH32	At3g25710	BASIC HELIX-LOOP-HELIX 32 transcription factor, ABA-regulated protein AIG1, TARGET OF MONOPTEROS 5, TMO5	Chen *et al.* (2007)	CACAACCAAAACGGACAAAGC	59.1	ATCGCACTCTGTTGGGACTTG	60.6	intron too far away from 3' UTR	115	none	1.86 ± 0.01
FBX2	At5g21040	F-BOX PROTEIN 2, F-box containing protein that interacts physically with BHLH32	Chen *et al.* (2008a)	AATGCTCCATTGCTGCTCATAA	58.7	CCCGCTTGCTCTTCGTTTAC	59.6	intron too far away from 3' UTR	80	same as cDNA	1.80 ± 0.01
G3PP1	At3g47420	GLYCEROL-3-PHOSPHATE PERMEASE 1, PHOSPHATE STARVATION-INDUCED GENE 3, PS3	Ramaiah *et al.* (2011)	GTTTGTGGCTGGACTATTGTTGAC	60.6	GTCCACCGGAGATCTACATTCTTC	60.5	intron too far away from 3' UTR	100	same as cDNA	1.84 ± 0.02
IPS1	At3g09922	INDUCED BY PHOSPHATE STARVATION1, A. THALIANA INDUCED BY PHOSPHATE STARVATION1, ATIPS1	Martin *et al.* (2000)	GGGATGGCCTAAATACAAAATGAA	57.5	TCCATATCTTAAAACGCTTTCCTTACA	59.1	no intron	80	same as cDNA	1.86 ± 0.02
MIR399d	At2g34202	MICRORNA399D, Mature microRNA sequence: UGCCAAAGGAGAUUUGCCCCG	Bari *et al.* (2006)	GGTTGGATTACTGGGCGAATACT	60.4	CTCCTTTGGCAGAGAAGCATTTT	59.7	no intron	80	same as cDNA	1.83 ± 0.01
NMT3	At1g73600	N-METHYLTRANSFERASE 3, S-adenosyl-L-methionine-dependent phosphoethanolamine N-methyltransferase	Cruz-Ramirez *et al.* (2004)	GCACCAAGAAGGAGTATCCTGATAA	60.2	TGCTGGCTTGTCTTGGATATGT	60.0	3rd last intron	83	none	1.89 ± 0.01
PAP1	At1g13750	PURPLE ACID PHOSPHATASE 1, PAP1	Li *et al.* (2002)	CCGGTTTACCAGAGTGTATGCA	60.4	TTGAAGATCAGAGAATTCAGCAAGA	58.5	last intron	123	none	1.79 ± 0.02
PHF1	At3g52190	PHOSPHATE TRANSPORTER TRAFFIC FACILITATOR1, related to the SEC12 proteins of the early secretory pathway	Gonzalez *et al.* (2005)	AGTGGAAAGAGTGGCAGATATATGC	60.7	CTCGAAGAATACGTATGCAGCTATCA	60.8	last intron	80	none	1.72 ± 0.02
PHO1	At3g23430	PHOSPHATE 1, SPX domain containing protein	Hamburger *et al.* (2002)	GGGTCAGTCCTGTTCAGTCTCAT	61.6	GTGCTCATTCTCCACTCTGTAAAAGT	61.2	last intron	101	none	1.79 ± 0.02
PHO1;H1	At1g68740	PHOSPHATE 1 family member H1, SPX domain containing protein	Stefanovic *et al.* (2007)	TACCGATTGGAGAATGAGCATCTAA	59.7	TTAGTCTTCTTCATCCACTTCTCTGAAAG	60.9	last intron	96	none	1.80 ± 0.02
PHO2	At2g33770	PHOSPHATE 2, UBIQUITIN-CONJUGATING ENZYME 24, UBC24	Bari *et al.* (2006)	CGAGCCGAGGGAGAGAAAAA	59.8	CATCTCAAAATGCTTTGGAGGCT	59.8	3rd last intron	103	none	1.80 ± 0.02
PHR1	At4g28610	PHOSPHATE STARVATION RESPONSE 1, MYB transcription factor	Rubio *et al.* (2001)	ATATCGGCCAGAACCATCAGAAAC	61.0	TGTAATACCTATCCCACCTTTCAAATC	59.0	2nd last intron	100	none	1.89 ± 0.01
PHT1;4	At2g38940	PHOSPHATE TRANSPORTER 1;4, ARABIDOPSIS THALIANA PHOSPHATE TRANSPORTER 2, ATPT2	Shin *et al.* (2004)	TGTGCCGGCCGAAATCT	59.3	TTGCTCCTAATTTTCCTGATGCT	58.4	intron too far away from 3' UTR	80	same as cDNA	1.84 ± 0.02
PHT1;7	At3g54700	PHOSPHATE TRANSPORTER 1;7	Müller *et al.* (2004)	CGCGGCTTCTGGAAAATTAG	57.5	TGGAGGATATCCATGCTCTGTCT	60.4	no intron	100	same as cDNA	1.83 ± 0.03
PLDz2	At3g05630	PHOSPHOLIPASE D ZETA 2	Cruz-Ramirez *et al.* (2006)	CACGCCGGAGAGATTCAGA	59.2	TGTCCGTGTTTTTCTTAGCTGTTG	60.2	2nd intron	91	none	1.86 ± 0.02
PPCK2	At3g04530	PHOSPHOENOLPYRUVATE CARBOXYLASE KINASE 2, PEPCK2	Chen *et al.* (2008b)	GCAGAGGATGCTCTTCGACATT	60.7	CGAATCAAACAATTACACACTCACAT	58.9	last intron	141	none	1.86 ± 0.01
RNS1	At2g02990	RIBONUCLEASE 1, member of the ribonuclease T2 family, responds to inorganic phosphate starvation, and inhibits production of anthocyanin	Bariola *et al.* (1994)	CTCTAACCAAAGCCGGGATTAA	58.1	ACCAATTGACTCTTTTATCGAATCTCT	59.0	last intron	83	none	1.88 ± 0.01
SPX1	At5g20150	SPX (yeast Syg1, Pho81, and the human XPR1 protein) DOMAIN GENE 1	Duan *et al.* (2008)	CAATTGTTAGAAGACGAGTTGGAGAA	59.6	CCATTGAATCCTTAGCTTTCGCA	59.6	spans first intron	118	200	1.78 ± 0.01
SQD2	At5g01220	SULFOQUINOVOSYLDIACYLGLYCEROL 2, UDP-sulfoquinovose:DAG sulfoquinovosyltransferase	Yu *et al.* (2002)	CCTTGTGGTTCTTGAAGCAATGT	59.9	ATCCGGTTTTTCCCTCCTGATC	60.1	last intron	104	none	1.86 ± 0.01
**new P signaling candidates (selected from published micro arrays in Genevestigator)**											
C3HC4	At5g19430	C3HC4-type RING finger E3 ubiquitin ligase, RING/U-box superfamily	Misson *et al.* (2005)	TTGGGCGAGTTGCAAAGAG	58.7	AAAATCTTCAATGCTGCCATCA	57.2	last intron	100	none	1.79 ± 0.03
PUB35	At4g25160	PLANT U-BOX protein 35, U-box domain-containing E3 ubiquitin ligase with central Ser/Thr protein kinase domain	Wiborg *et al.* (2008)	GGGAAAGATAGACCTGATTTGAAAGA	58.9	GCTCGTTCATCACGTCCTTAAGT	60.9	last intron	154	none	1.86 ± 0.02
UBC25	At3g15355	UBIQUITIN-CONJUGATING ENZYME 25, PHO2 FAMILY UBIQUITIN CONJUGATION ENZYME 1, PFU1	Eifler, K. - PhD thesis (2010)	ATGAGGAGACCACCCAAGTATTTT	59.7	CCGGTGTAGCTCCGTTTCTG	60.7	last intron	109	none	1.85 ± 0.01

References

Bari R, Pant BD, Stitt M, Scheible WR. 2006. PHO2, microRNA399, and PHR1 define a phosphate-signaling pathway in plants. *Plant Physiology* **141**, 988-999.

Bariola PA, Howard CJ, Taylor CB, Verburg MT, Jaglan VD, Green PJ. 1994. The Arabidopsis ribonuclease gene *RNS1* is tightly controlled in response to phosphate limitation. *Plant Journal* **6**, 673-685.

Chen ZH, Jenkins GI, Nimmo HG. 2008a. Identification of an F-Box protein that negatively regulates P_i starvation responses. *Plant and Cell Physiology* **49**, 1902-1906.

Chen ZH, Jenkins GI, Nimmo HG. 2008b. pH and carbon supply control the expression of phosphoenolpyruvate carboxylase kinase genes in *Arabidopsis thaliana*. *Plant, Cell & Environment* **31**, 1844-1850.

Chen ZH, Nimmo GA, Jenkins GI, Nimmo HG. 2007. bHLH32 modulates several biochemical and morphological processes that respond to Pi starvation in Arabidopsis. *Biochemical Journal* **405**, 191-198.

Cruz-Ramirez A, Lopez-Bucio J, Ramirez-Pimentel G, Zurita-Silva A, Sanchez-Calderon L, Ramirez-Chavez E, Gonzalez-Ortega E, Herrera-Estrella L. 2004. The *xipotl* mutant of Arabidopsis reveals a critical role for phospholipid metabolism in root system development and epidermal cell integrity. *Plant Cell* **16**, 2020-2034.

Cruz-Ramirez A, Oropeza-Aburto A, Razo-Hernandez F, Ramirez-Chavez E, Herrera-Estrella L. 2006. Phospholipase Dz2 plays an important role in extraplastidic galactolipid biosynthesis and phosphate recycling in Arabidopsis roots. *Proceedings of the National Academy of Sciences of the United States of America* **103**, 6765-6770.

Czechowski T, Stitt M, Altmann T, Udvardi MK, Scheible WR. 2005. Genome-wide identification and testing of superior reference genes for transcript normalization in Arabidopsis. *Plant Physiology* **139**, 5-17.

del Pozo JC, Allona I, Rubio V, Leyva A, de la Pena A, Aragoncillo C, Paz-Ares J. 1999. A type 5 acid phosphatase gene from *Arabidopsis thaliana* is induced by phosphate starvation and by some other types of phosphate mobilising/oxidative stress conditions. *Plant Journal* **19**, 579-589.

Duan K, Yi KK, Dang L, Huang HJ, Wu W, Wu P. 2008. Characterization of a sub-family of Arabidopsis genes with the SPX domain reveals their diverse functions in plant tolerance to phosphorus starvation. *Plant Journal* **54**, 965-975.

Eifler K. 2010. The PHO2 family of ubiquitin conjugating enzymes in *Arabidopsis thaliana* and its contribution to plant programmed cell death, University of Cologne, Cologne, 192.

Gonzalez E, Solano R, Rubio V, Leyva A, Paz-Ares J. 2005. PHOSPHATE TRANSPORTER TRAFFIC FACILITATOR1 is a plant-specific SEC12-related protein that enables the endoplasmic reticulum exit of a high-affinity phosphate transporter in Arabidopsis. *Plant Cell* **17**, 3500-3512.

Hamburger D, Rezzonico E, Petetot JMC, Somerville C, Poirier Y. 2002. Identification and characterization of the Arabidopsis *PHO1* gene involved in phosphate loading to the xylem. *Plant Cell* **14**, 889-902.

Li DP, Zhu HF, Liu KF, Liu X, Leggewie G, Udvardi M, Wang DW. 2002. Purple acid phosphatases of *Arabidopsis thaliana* - comparative analysis and differential regulation by phosphate deprivation. *Journal of Biological Chemistry* **277**, 27772-27781.

Martin AC, del Pozo JC, Iglesias J, Rubio V, Solano R, de la Pena A, Leyva A, Paz-Ares J. 2000. Influence of cytokinins on the expression of phosphate starvation responsive genes in Arabidopsis. *Plant Journal* **24**, 559-567.

Misson J, Raghothama KG, Jain A, Jouhet J, Block MA, Bligny R, Ortet P, Creff A, Somerville S, Rolland N, Doumas P, Nacry P, Herrerra-Estrella L, Nussaume L, Thibaud MC. 2005. A genome-wide transcriptional analysis using *Arabidopsis thaliana* Affymetrix gene chips determined plant responses to phosphate deprivation. *Proceedings of the National Academy of Sciences of the United States of America* **102**, 11934-11939.

Müller R, Nilsson L, Krintel C, Nielsen TH. 2004. Gene expression during recovery from phosphate starvation in roots and shoots of *Arabidopsis thaliana*. *Physiologia Plantarum* **122**, 233-243.

Ramaiah M, Jain A, Baldwin JC, Karthikeyan AS, Raghothama KG. 2011. Characterization of the phosphate starvation-induced glycerol-3-phosphate permease gene family in Arabidopsis. *Plant Physiology* **157**, 279-291.

Rubio V, Linhares F, Solano R, Martin AC, Iglesias J, Leyva A, Paz-Ares J. 2001. A conserved MYB transcription factor involved in phosphate starvation signaling both in vascular plants and in unicellular algae. *Genes & Development* **15**, 2122-2133.

Shin H, Shin HS, Chen R, Harrison MJ. 2006. Loss of *At4* function impacts phosphate distribution between the roots and the shoots during phosphate starvation. *Plant Journal* **45**, 712-726.

Shin H, Shin HS, Dewbre GR, Harrison MJ. 2004. Phosphate transport in Arabidopsis: Pht1;1 and Pht1;4 play a major role in phosphate acquisition from both low- and high-phosphate environments. *Plant Journal* **39**, 629-642.

Stefanovic A, Ribot C, Rouached H, Wang Y, Chong J, Belbahri L, Delessert S, Poirier Y. 2007. Members of the *PHO1* gene family show limited functional redundancy in phosphate transfer to the shoot, and are regulated by phosphate deficiency via distinct pathways. *Plant Journal* **50**, 982-994.

Wiborg J, O'Shea C, Skriver K. 2008. Biochemical function of typical and variant *Arabidopsis thaliana* U-box E3 ubiquitin-protein ligases. *Biochemical Journal* **413**, 447-457.

Yu B, Xu C, Benning C. 2002. Arabidopsis disrupted in *SQD2* encoding sulfolipid synthase is impaired in phosphate-limited growth. *Proceedings of the National Academy of Sciences of the United States of America* **99**, 5732-5737.

Supplemental Table S2: Time-course of relative transcript abundance of known phosphate-responsive genes in phosphorus-limited *A.thaliana* seedlings in response to either phosphate resupply or phosphite treatment. For *priMIR399d* and *PHT1;7* transcripts, suppression by phosphate was so strong that values were close to the detection limit (40-ΔCt value of 25.7 ± 0.1).

Roots	relative expression values in seedling roots expressed as 40 - DCt after normalisation against *PP2AA3* and *UBC9* reference genes																									
	P-sufficient control		P-limited roots								P_i-resupplied roots								Phi-treated roots							
			1 day		2 days		3 days		7 days		1 day		2 days		3 days		7 days		1 day		2 days		3 days		7 days	
GOI	mean	SE	mean	SE	mean	SE	mean	SE	mean	SE	mean	SE	mean	SE	mean	SE	mean	SE	mean	SE	mean	SE	mean	SE	mean	SE
ACP5	37.62	0.24	40.69	0.29	41.99	0.96	43.58	0.49	43.97	0.32	38.22	0.23	38.99	0.11	39.54	0.20	39.28	0.65	40.37	0.32	41.71	0.44	42.73	0.29	43.18	0.24
At4	41.11	0.31	45.45	0.33	46.41	0.48	47.15	0.27	47.44	0.23	44.10	0.09	42.77	0.34	41.97	0.34	41.98	0.78	45.05	0.11	46.10	0.07	46.75	0.14	46.65	0.19
BHLH32	37.76	0.21	37.37	0.37	37.46	0.22	37.32	0.15	37.58	0.21	37.11	0.35	37.30	0.34	37.13	0.38	37.14	0.30	36.96	0.24	37.86	0.14	37.53	0.07	37.45	0.43
C3HC4	37.14	0.12	37.73	0.22	38.05	0.49	38.99	0.25	39.25	0.32	37.20	0.09	36.97	0.10	37.21	0.56	36.99	0.30	37.58	0.26	37.54	0.15	38.55	0.45	38.25	0.17
FBX2	39.29	0.08	39.28	0.04	39.67	0.36	39.60	0.37	39.26	0.18	39.51	0.06	39.40	0.05	39.45	0.33	39.24	0.44	39.38	0.21	40.10	0.37	39.85	0.36	39.68	0.10
G3PP1	40.23	0.50	42.09	0.42	43.03	0.73	44.34	0.30	44.41	0.11	39.89	0.38	40.13	0.32	40.78	0.21	41.05	0.60	42.41	0.46	43.15	0.40	44.22	0.05	44.18	0.05
IPS1	34.99	0.23	41.83	0.50	43.50	1.09	45.21	0.60	46.34	0.08	40.15	0.14	38.57	0.72	37.15	0.10	36.06	0.23	41.79	0.48	42.99	0.34	44.28	0.56	44.60	0.28
pri-MIR399d	27.48	1.24	31.58	1.02	33.41	1.51	37.96	0.45	39.70	0.21	25.90	1.00	27.65	0.29	26.71	0.50	27.36	1.15	31.91	0.85	34.28	0.35	36.58	0.30	38.00	0.90
NMT3	35.98	0.28	34.29	0.76	34.23	0.38	33.88	0.29	33.50	0.32	35.88	0.23	34.97	0.39	34.95	0.69	37.19	0.23	35.47	0.31	35.47	0.54	35.36	0.06	33.67	0.29
PAP1	37.85	0.23	39.37	0.13	39.70	0.53	40.65	0.27	41.25	0.16	38.34	0.19	37.89	0.05	37.77	0.10	37.67	0.21	38.87	0.17	39.12	0.08	39.92	0.04	40.26	0.14
PHF1	39.24	0.25	40.15	0.20	40.83	0.37	41.02	0.10	40.96	0.06	39.32	0.18	38.58	0.45	38.85	0.09	38.68	0.23	40.35	0.26	39.66	0.44	40.62	0.27	40.04	0.06
PHO1	42.21	0.20	41.93	0.18	41.83	0.26	42.17	0.08	41.77	0.52	41.96	0.07	41.39	0.27	41.63	0.25	42.29	0.23	42.47	0.21	42.48	0.13	42.86	0.24	43.19	0.38
PHO1;H1	41.59	0.17	42.02	0.23	42.32	0.77	44.16	0.27	43.64	0.46	41.75	0.29	41.10	0.30	41.67	0.24	40.91	0.47	41.88	0.29	42.01	0.27	43.41	0.13	43.04	0.25
PHO2	40.47	0.30	39.13	0.18	39.45	0.13	40.32	0.16	39.81	0.34	39.77	0.14	39.59	0.11	40.49	0.14	40.07	0.10	39.54	0.07	39.81	0.15	40.56	0.48	40.50	0.29
PHR1	37.57	0.37	37.50	0.15	37.36	0.32	37.15	0.32	37.24	0.14	37.38	0.04	37.29	0.12	37.37	0.24	37.23	0.28	37.05	0.35	37.42	0.18	37.21	0.54	37.36	0.21
Pht1;4	41.16	0.50	42.74	0.24	42.67	1.12	44.12	0.35	44.62	0.11	40.82	0.22	40.35	0.64	40.53	0.42	40.55	0.44	42.62	0.25	42.57	0.67	43.25	0.45	43.13	0.22
Pht1;7	28.91	0.72	30.02	0.25	34.42	2.23	35.79	0.81	37.80	0.38	26.59	1.86	28.87	2.18	27.59	0.82	25.64	0.94	30.13	2.45	32.16	1.19	33.99	0.05	33.39	0.35
PLDz2	33.81	0.16	35.66	0.25	36.42	1.00	38.15	0.40	39.22	0.17	34.30	0.08	33.34	0.52	33.45	0.46	33.45	0.31	35.85	0.18	36.50	0.54	37.85	0.34	38.57	0.14
PPCK2	37.39	0.35	37.75	0.12	38.35	0.25	39.42	0.22	40.68	0.28	36.89	0.44	37.13	0.47	37.84	0.17	36.42	1.03	36.97	0.15	38.20	0.19	38.93	0.05	39.92	0.48
PUB35	33.00	0.32	34.17	0.34	35.36	0.27	36.61	0.58	37.44	0.35	33.62	0.30	33.35	0.19	34.37	0.64	33.50	0.21	34.15	0.16	34.03	0.15	35.41	0.55	34.91	0.09
RNS1	34.82	0.57	37.12	0.45	37.82	1.15	39.52	1.00	40.68	0.17	36.10	0.42	36.16	0.51	35.90	0.66	35.87	0.30	37.27	0.44	38.07	0.25	38.42	0.08	38.26	0.24
SPX1	37.13	0.56	39.17	0.28	40.96	0.44	42.27	0.27	42.49	0.27	36.19	0.34	34.89	0.14	34.52	0.23	36.09	0.43	38.37	0.49	40.32	0.16	41.11	0.30	41.11	0.18
SQD2	35.99	0.39	37.47	0.29	38.01	0.75	41.51	0.20	41.30	0.10	36.07	0.35	35.72	0.03	36.07	0.19	35.56	0.07	36.10	0.15	36.67	0.12	38.43	0.38	38.98	0.18
UBC25	39.25	0.18	39.49	0.14	39.53	0.08	39.65	0.05	40.01	0.15	39.26	0.24	39.64	0.24	39.37	0.12	39.71	0.15	39.38	0.14	40.04	0.06	39.99	0.07	39.59	0.09

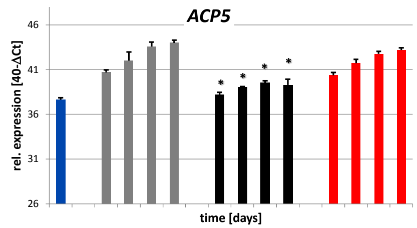

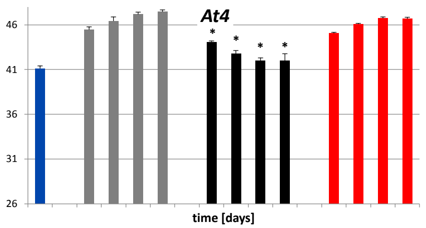

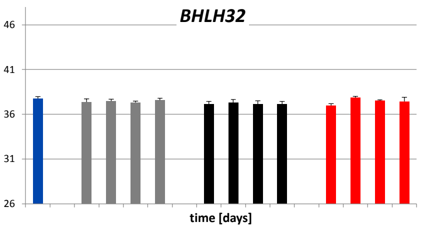

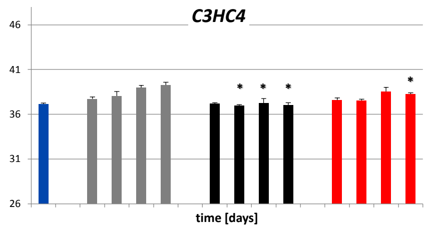

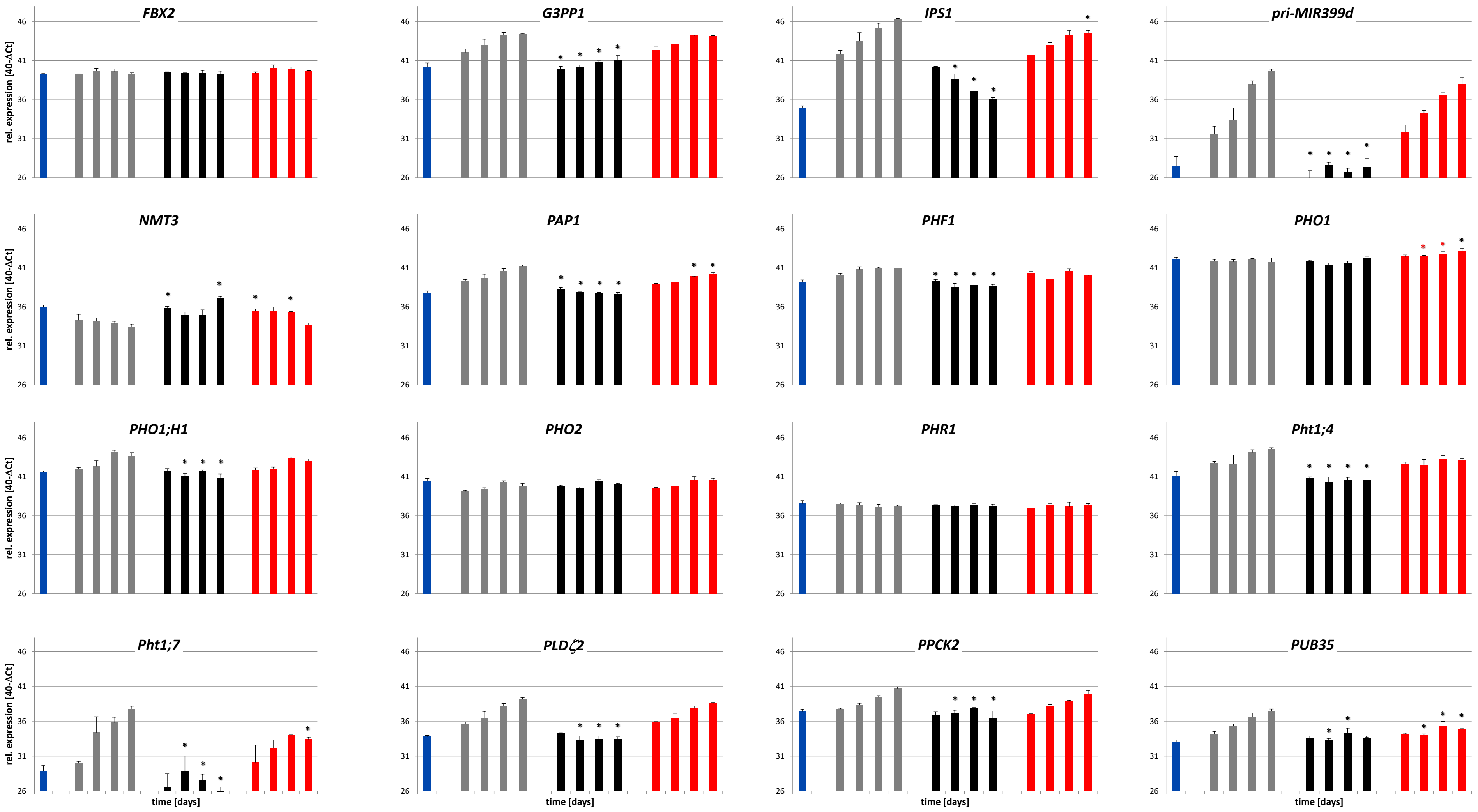
FBX2
G3PP1
IPS1
pri-MIR399d
NMT3
PAP1
PHF1
PHO1
PHO1;H1
PHO2
PHR1
Pht1;4
Pht1;7
PLDζ2
PPCK2
PUB35
rel. expression [40-ΔCt]
time [days]
46
41
36
31
26
*

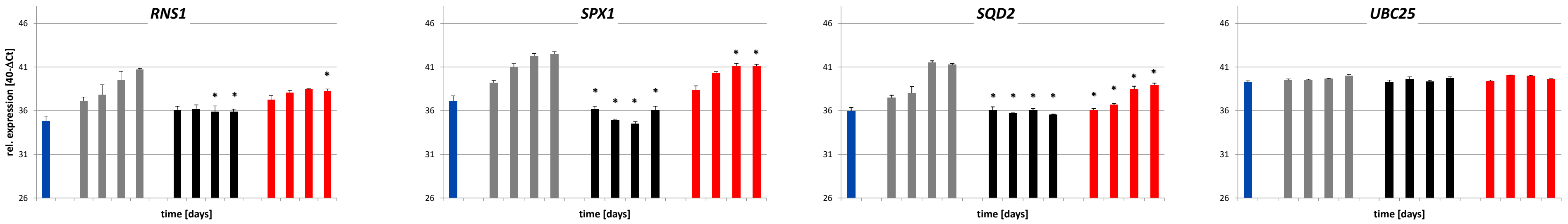

legend:

= phosphorus-sufficient control plant

= phosphorus-limited plant

= phosphate-resupplied plant

= phosphite-treated plant

Statistics:

2-WAY ANOVA + Tukey's Test

* = significant difference compared to phosphorus-limited roots for a given time point, $p \leq 0.05$

* = significant difference between phosphite-treated and phosphate-resupplied, but not P-limited and phosphate-resupplied roots, for a given time point, $p \leq 0.05$

Shoots	relative expression values in seedling shoots expressed as 40 - ΔCt after normalisation against *PP2AA3* and *UBC9* reference genes																									
	P-sufficient control		P-limited roots								P_i-resupplied roots								Phi-treated roots							
			1 day		2 days		3 days		7 days		1 day		2 days		3 days		7 days		1 day		2 days		3 days		7 days	
GOI	mean	SE	mean	SE	mean	SE	mean	SE	mean	SE	mean	SE	mean	SE	mean	SE	mean	SE	mean	SE	mean	SE	mean	SE	mean	SE
ACP5	37.44	0.28	39.78	0.47	40.11	0.69	41.84	0.37	42.79	0.26	36.64	0.92	37.30	0.32	38.30	0.58	37.89	0.64	39.25	0.30	39.79	0.21	40.87	0.25	41.53	0.29
At4	37.78	0.19	42.10	0.18	43.55	0.13	44.88	0.49	46.31	0.14	41.27	0.50	41.48	0.10	39.57	0.13	38.87	0.52	42.42	0.44	42.76	0.10	44.00	0.17	44.34	0.06
BHLH32	33.83	0.44	34.20	0.66	35.07	0.36	34.36	0.12	35.85	0.20	33.21	0.30	34.70	0.46	33.83	0.33	34.41	0.08	34.34	0.11	35.36	0.50	34.79	0.24	33.93	0.53
C3HC4	37.57	0.28	38.28	0.24	38.57	0.28	38.78	0.17	39.61	0.04	37.67	0.28	38.11	0.05	37.63	0.38	37.73	0.25	38.31	0.32	38.15	0.12	38.33	0.49	37.71	0.27
FBX2	39.82	0.27	39.26	0.62	39.92	0.08	40.36	0.10	40.20	0.09	39.65	0.66	40.02	0.46	39.47	0.21	39.76	0.10	39.38	0.49	40.03	0.14	40.11	0.21	40.07	0.11
G3PP1	40.45	1.11	41.51	0.76	41.53	0.52	42.70	0.18	43.30	0.26	39.64	1.17	39.55	0.21	41.12	0.17	40.11	0.60	41.01	0.47	41.02	0.03	41.63	0.13	41.98	0.24
IPS1	36.09	0.64	41.17	0.21	42.16	0.32	43.15	0.50	44.34	0.34	39.44	0.18	38.79	0.26	36.65	0.10	34.46	0.23	41.36	0.26	41.11	0.17	41.39	0.20	40.73	0.43
pri-MIR399d	29.53	0.20	36.08	0.45	36.35	1.21	39.93	1.36	41.26	0.42	28.22	0.31	29.12	2.06	29.13	0.77	31.30	0.73	34.85	0.80	34.38	0.60	37.58	0.51	38.33	0.65
NMT3	36.96	0.66	36.12	0.13	36.29	0.40	35.81	0.20	35.01	0.31	36.27	0.32	36.45	0.28	36.59	0.94	36.93	0.22	36.84	0.35	37.26	0.35	36.78	0.34	35.09	0.74
PAP1	36.99	0.33	38.25	0.39	38.79	0.35	39.37	0.37	41.35	0.29	37.22	0.34	37.57	0.14	36.78	0.39	38.29	0.34	39.19	0.26	38.49	0.24	39.47	0.30	40.61	0.18
PHF1	38.79	0.43	39.73	0.37	39.20	0.29	39.74	0.23	39.71	0.27	39.06	0.19	38.21	0.13	38.36	0.36	37.84	0.14	39.96	0.47	38.79	0.24	38.72	0.41	38.73	0.14
PHO1	37.18	0.62	37.47	0.58	38.05	0.65	38.43	0.15	39.12	0.06	37.05	0.58	37.49	0.20	37.57	0.22	36.31	0.33	37.91	0.34	38.41	0.06	38.92	0.24	38.83	0.42
PHO1;H1	39.05	0.38	39.58	0.40	41.34	0.70	42.32	0.26	42.62	0.14	38.05	0.53	38.79	0.12	37.64	1.24	38.47	0.61	39.93	0.17	39.99	0.18	41.69	0.17	40.45	0.33
PHO2	40.00	0.60	39.81	0.59	38.77	0.44	39.50	0.39	40.15	0.28	39.01	0.37	38.11	0.12	38.57	0.23	38.15	0.13	39.92	0.59	38.63	0.08	38.90	0.27	39.12	0.18
PHR1	36.96	0.44	37.00	0.46	38.79	0.08	38.70	0.30	38.19	0.14	38.19	0.30	38.57	0.23	37.91	0.30	38.36	0.32	38.07	0.43	38.65	0.13	37.96	0.53	37.95	0.20
Pht1;4	40.75	0.76	41.25	0.78	38.88	0.78	39.93	0.52	41.56	0.26	39.64	0.98	37.20	0.16	37.21	0.33	35.58	0.21	42.56	0.33	39.00	0.42	39.95	0.43	40.19	0.10
Pht1;7	27.73	1.83	34.53	1.50	32.18	2.13	34.05	0.93	35.77	0.30	27.72	1.83	26.23	0.87	26.57	0.89	28.37	0.94	31.72	1.35	28.10	1.40	33.05	1.18	34.53	0.61
PLD ζ2	35.78	1.56	35.84	0.91	35.88	0.90	37.62	0.45	38.35	0.35	34.92	1.29	33.43	0.72	33.62	0.55	34.44	0.50	37.07	1.65	35.61	0.20	36.62	0.37	36.71	0.20
PPCK2	35.87	0.14	38.01	0.81	34.71	0.55	37.31	0.33	39.92	0.69	32.92	1.53	31.80	0.51	31.80	1.05	32.33	1.22	37.38	1.10	35.26	0.13	36.46	0.49	37.37	0.60
PUB35	32.71	1.44	33.40	1.08	31.25	0.27	33.02	0.43	33.38	0.38	32.80	1.26	30.14	0.04	29.95	0.40	29.75	0.25	32.10	1.74	30.09	0.42	30.63	0.58	31.24	0.65
RNS1	35.21	0.25	37.45	0.32	35.42	1.81	37.71	0.55	39.74	0.25	35.55	0.28	34.78	1.32	36.00	0.23	33.73	0.65	35.29	1.00	35.76	0.78	37.52	1.06	37.77	0.05
SPX1	35.34	0.38	37.59	0.46	37.24	0.20	38.54	0.31	40.04	0.22	34.55	0.42	34.37	0.43	35.43	0.43	34.85	0.53	37.55	0.44	37.03	0.18	36.85	0.16	37.48	0.18
SQD2	36.62	0.43	38.07	0.46	38.87	0.50	41.05	0.87	41.18	0.01	36.61	0.61	36.59	0.33	37.40	0.34	37.40	0.18	37.72	0.40	38.02	0.23	38.99	0.18	38.05	0.07
UBC25	39.39	0.14	38.60	0.48	40.03	0.12	39.77	0.19	39.57	0.49	38.75	0.62	39.78	0.25	39.59	0.05	40.13	0.38	38.86	0.51	39.87	0.01	39.68	0.09	40.20	0.19

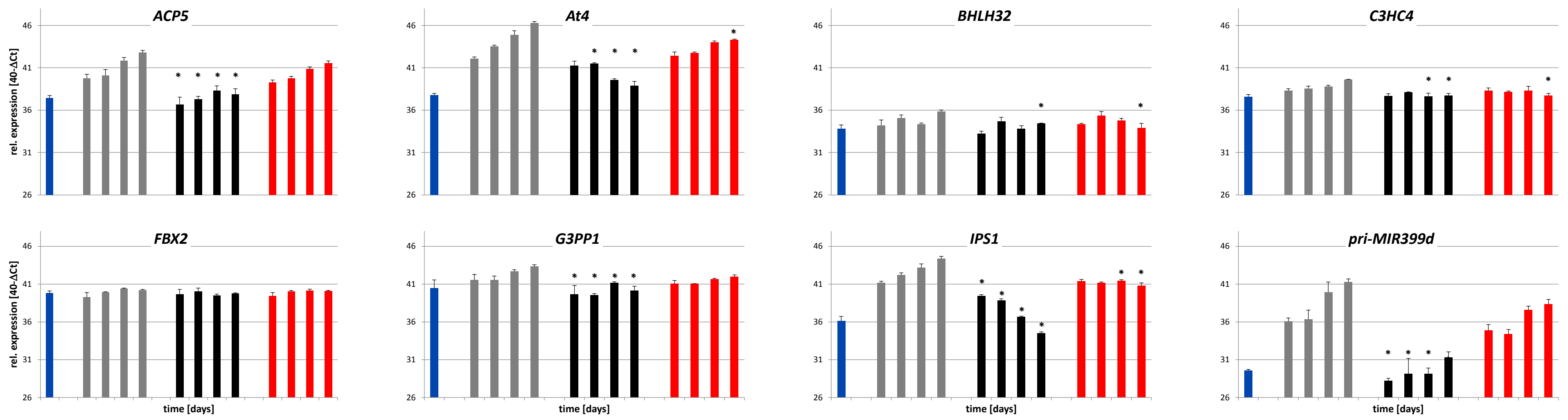

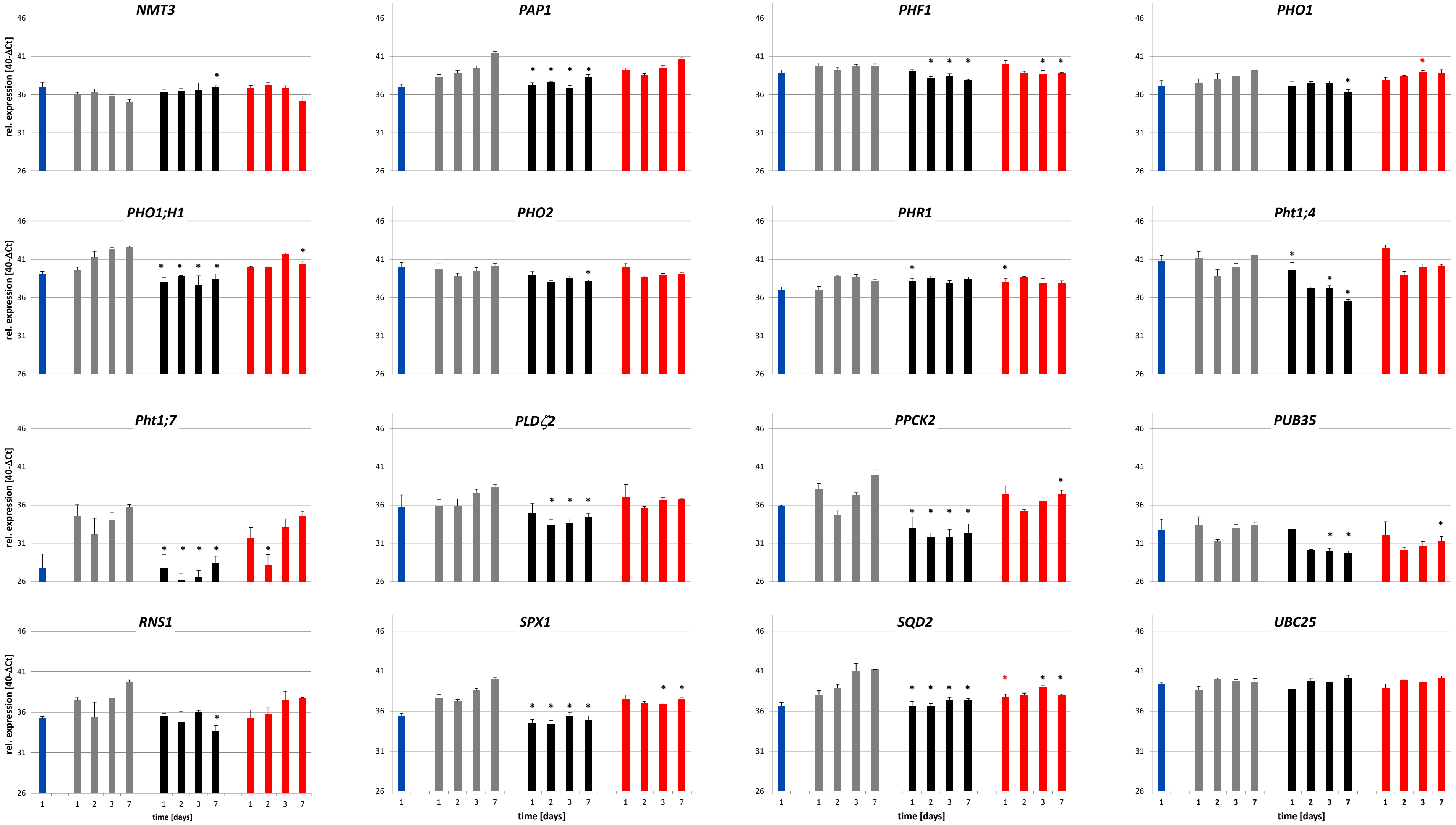
NMT3
PAP1
PHF1
PHO1
PHO1;H1
PHO2
PHR1
Pht1;4
Pht1;7
PLDζ2
PPCK2
PUB35
RNS1
SPX1
SQD2
UBC25
rel. expression [40-ΔCt]
time [days]

legend:

■ = phosphorus-sufficient control plant

■ = phosphorus-limited plant

■ = phosphate-resupplied plant

■ = phosphite-treated plant

Statistics:

2-WAY ANOVA + Tukey's Test

* = significant difference compared to phosphorus-limited roots for a given time point, $p \leq 0.05$

* = significant difference between phosphite-treated and phosphate-resupplied, but not P-limited and phosphate-resupplied roots, for a given time point, $p \leq 0.05$

Supplemental Figure S1:

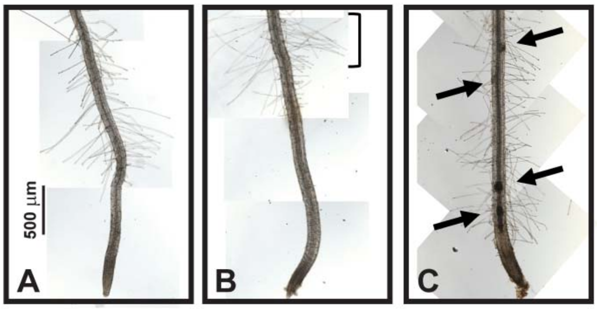

<u>Supplemental Figure S1:</u> Root phenotypic responses in phosphorus-limited plants to phosphate (P_i) resupply or phosphite (Phi) treatment. For this experiment, plants were grown hydroponically in an agar-free version of the nutrient solution used for the plate experiments (see Materials and Methods). Four-week old plants were deprived of P_i for 12 days before being resupplied with nutrient solution containing 250 µM P_i or treated with minimal nutrient solution containing 1 mM Phi. Nutrient solutions were replaced daily before microscopic images of roots were taken on day 5. (A) Primary root of a control plant that was deprived of P_i for a total of 17 days. (B) Root of a plant resupplied with P_i for five days, still featuring long root hairs in the segments that were formed prior to the treatment (bracket). (C) Root of a Phi-treated plant showing the typical growth arrest of the primary root as well as lateral roots (arrows). The scale is the same for all panels.

Supplemental Figure S2:

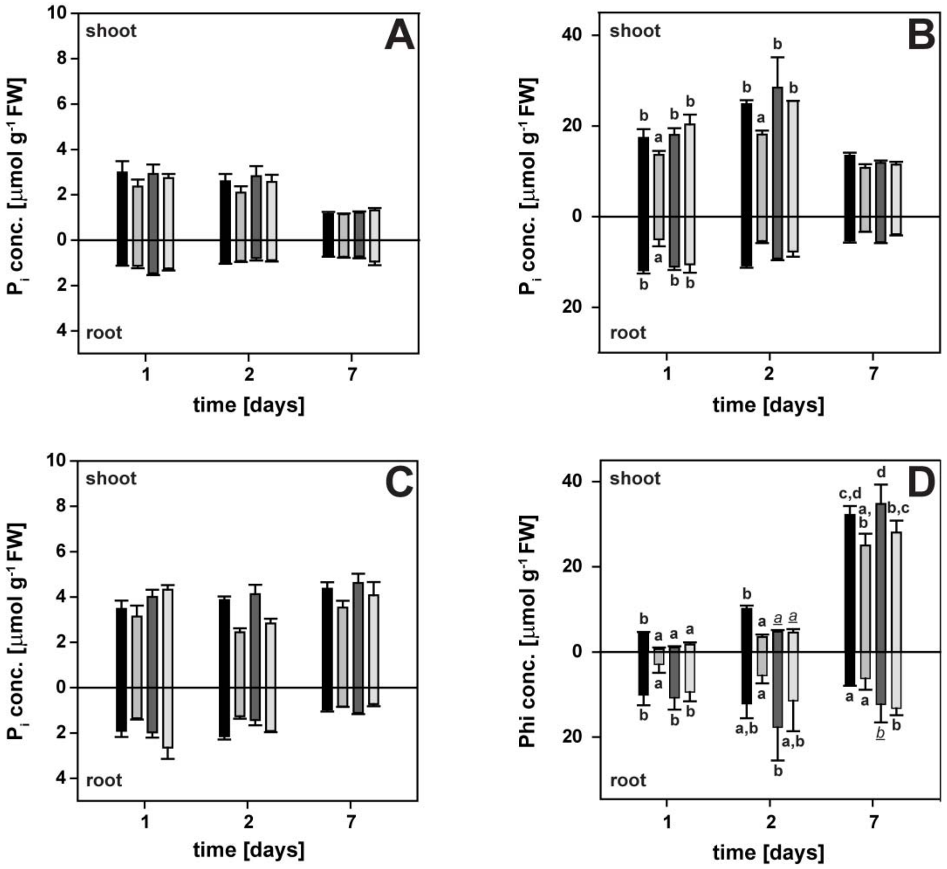

Supplemental Figure S2: Phosphate (P_i) and phosphite (Phi) accumulation in roots and shoots of Col-0 and *pht1* mutants over time. Five-day old seedlings were depleted of P_i for four days before treatment. Root and shoot accumulation of P_i after (A) P-limitation, (B) P_i resupply, (C) Phi treatment. (D) Phi accumulation in roots and shoots of Phi-treated seedlings. Shown are means ± SE, n = 3 plates with 12 seedlings each. There is no significant difference between genotypes and treatments for each organ and time point when they share a letter, according to Tukey's HSD at $p < 0.05$ or $p < 0.1$ (underlined, italic letters). Genotypes tested were Col-0 (black bars), *pht1;1-2* (grey bars), *pht1;8* (dark grey bars) and *pht1;9-1* (light-grey bars).

Supplemental Figure S3:

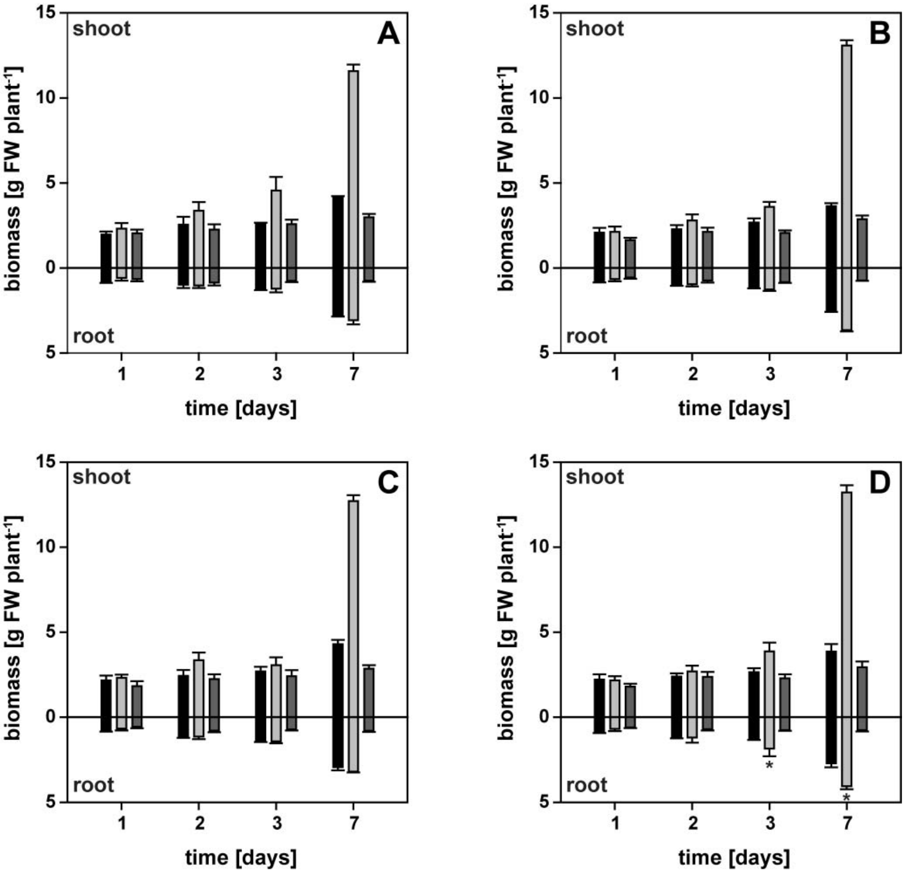

Supplemental Figure S3: Biomass accumulation in roots and shoots of Col-0 and *pht1* mutants over time. Five-day old seedlings were depleted of phosphate (P_i) for four days and either grown without P_i for another seven days (black bars), treated with 250 µM P_i (light grey bars) or treated with 250 µM phosphite (dark grey bars). Root and shoot biomass was determined for (A) Col-0, (B) *pht1;1-2*, (C) *pht1;8* and (D) *pht1;9-1*. Shown are means ± SE, n = 3 plates with 12 seedlings each. Statistically significant differences between genotypes within each organ and treatment over time are indicated by * according to Tukey's HSD at $p < 0.05$.
